# DeTi: A Decentralized Ticketing Management Platform

**DOI:** 10.1007/s10922-022-09675-3

**Published:** 2022-07-26

**Authors:** Sina Rafati Niya, Simon Bachmann, Claudio Brasser, Michael Bucher, Nicolas Spielmann, Burkhard Stiller

**Affiliations:** Communication Systems Group CSG, Department of Informatics IfI, University of Zürich UZH, Binzmühlestrasse 14, 8050 Zürich, Switzerland

**Keywords:** Blockchain, Smart contracts, Decentralized applications, Decentralized ticketing, Distributed service management

## Abstract

Event tickets being sold in their electronic instances are subject to counterfeiting, profiteering, and black markets. Therefore, suitable service management mechanisms are required to overcome such deficits. This work designs, develops, and evaluates the approach of a Decentralized Ticketing platform—called DeTi—for managing the distribution of electronic event tickets and “regulating” the aftermarket. DeTi offers a dedicated service management functionality by operating through Smart Contracts of Ethereum, such that users can verify tickets’ validity for a given event. Especially, a new mechanism for users to detect fraudulent events is introduced, too. The evaluation performed indicates that DeTi invalidates or validates tickets efficiently via its decentralized and BC-based service management approach. By securing technically a set of underlying processes, DeTi obviates forging, replication, and scalping of tickets, allowing for a well-managed resale ecosystem of tickets based on and limited to the organizers’ initial pricing.

## Introduction

**Fig. 1:**
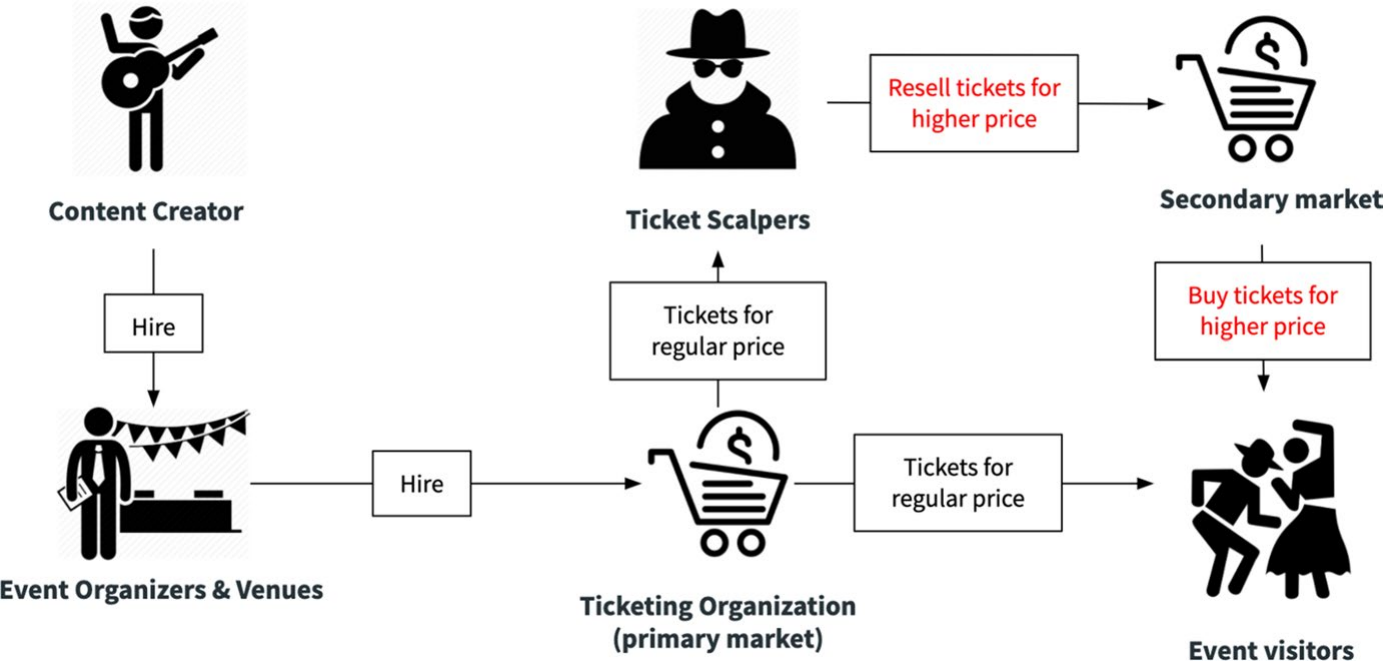
Actors in the ticketing industry [19]

Today’s event ticketing industry has fundamental flaws. Existing systems for distributing tickets are open for arbitrage during the time the ticket is sold until the event happens. For example, Ed Sheeran’s “Benefit Concert” at Albert Hall (UK) in 2017 for the Teenage Cancer Trust was abused for heavy profiteering, where tickets with a fee value of 75 £ have been resold for up to 2330 £ [1]. Beyond the reputation and fairness concerns, the black market is also problematic due to fraud related to the ticket themselves. Tickets are often fake or have already been used, rendering them invalid. This means that besides the primary seller of the tickets (e.g., event organizer) intermediaries make a profit by buying, reselling, and counterfeiting tickets. Figure 1 illustrates today’s event ticketing landscape with its key actors and their interactions. Here, event organizers are “Hosts”. Event visitors are “Guests”, and the black market determines the concerns with respect to fraud, high reselling prices, and ticket scalpers scooping profits through it.

To tackle such major concerns, different centralized ticketing management services have been introduced in the past. However, since centralized approaches inherit the key deficit of being a single point of failure, this work here focuses on decentralized solutions for the management of ticketing, such as offering a Blockchain (BC)-based service management functionality on the application layer. BCs are distributed ledgers, which store data via a network of distributed nodes [2]. BCs establish a tamper-proof ecosystem by its nodes, i.e., miners. All users, i.e., BC clients, show equal write and read rights. Miners perform a set of actions to mine blocks of Transactions (TX). These actions are specified by the consensus mechanism defined for that BC. Various consensus mechanisms exist and are used by different BCs, such as Proof-of-Work (PoW) in Bitcoin and Ethereum, Proof-of-Stake (PoS) in Cardano and Algorand, or Byzantine Fault Tolerant (BFT) and its variations in Hyperledger, Ripple, and Stellar [3].

In the BC realm, the Ethereum BC has distinguished itself by enabling distributed autonomous computation, while enabling data storage in a distributed setting, too [2]. Ethereum started by a proprietary PoW-based consensus, but with higher scalability than Bitcoin. Ethereum introduced Smart Contracts (SC) as distributed applications developed like programs, i.e., written by programming languages, but run in a decentralized manner and accessing in a decentral manner data stored. The invention of SCs has been a linchpin for decentralized management and computation of many applications, which led to the emergence of many services [4], including a decentralized ticketing management. BCs bring trust and transparency by their underlying cryptographic structure. However, such a decentralization, while preserving data with a high level of security, comes with various costs. For instance, PoW-based BCs suffer from scalability concerns with respect to low TX validation rates [5]. There exists also the user privacy concern with respect to the data being publicly available and not deletable. Another concern with using BCs is the cost of data storage.

The research questions to be answered in this paper are formulated as follows. (i) What are the requirements of ticketing platforms in order to prevent fraud and black markets. (ii) How BCs and SCs can be employed for addressing such requirements in managing presale, aftermarket, and identification processes. Therefore, this work studies user requirements to be addressed via a potentially reliable and secure ticketing platform, and collects key concerns experienced with existing ticketing platforms. Based on the collected information, this work proposes a novel decentralized ticketing management platform termed DeTi (Decentralized Ticketing Management Platform). DeTi approach’s novelty is briefed as follows.

- A *presale logic* which prevents front-running attacks and will not lead to sudden surges in gas prices and guarantees a fair distribution of tickets if the demand for an event is higher than its supply via a *transparent and publicly available lottery approach*.
- A *trustless queue-based approach* for aftermarket that enables buyers and sellers to transact with each other. Ticket scalpers have no scope of action in DeTi and they are prohibited on protocol level. Thus no black market is possible.
- Tickets *cannot* be sold for higher prices than their original price.
- Enabling high flexibility in identity management such as incentivizing interested parties to act as *Identity Approvers*, whom would be used to identify guests.
- Enabling an economic incentive management that considers all the stakeholders in the ticketing business.
- Employing *social trust certificates* to create a decentralized layer of trust for higher trust than offered in current ticketing platforms.

The remainder of this paper is organized as follows. User requirements and deficits of current ticketing systems are discussed in Sect. 2 followed by Sects. 3 to 3.11 covering design specifications and implementation details of DeTi. Section 4 discusses the economic incentive management of DeTi. Section 5 discusses evaluation results, while covering a comparison of the related work and DeTi. Finally, Sect. 6 provides a summary of achieved goals.

## Background and Related Work

In an event where a ticket generating and management system is needed, stakeholders can be classified into different groups. The following briefly overviews 5 of these potential groups and their role, covering their requirements in a ticketing system.

### Ticketing Ecosystem Stakeholders

(i) Guests: are the event attendees who buy the tickets from a ticketing system owned or contracted by the event Host. Guests want to be able to buy tickets for a fair and fixed price and, if needed, resell their owned tickets. If guests had to buy tickets from a different source than the event host, they need to be insured that the ticket they bought is valid and not a counterfeit. In the current ticketing landscape, the initial sale of tickets is not strictly regulated. Thus, scalpers can buy tickets in a bunch and resell them on the “aftermarket” for a higher price, i.e., creating a black market and making it hard for legit guests to obtain tickets when there is a high demand for that event. Furthermore, the validity of the tickets sold on the aftermarket cannot be verified easily.
(ii) Hosts: are entities that initiate and organize events. They are responsible for making deals with content creators and carry out events. Therefore, event Hosts issue tickets and sell them for pre-defined prices to event guests. In the current landscape, Hosts experience two main issues, (a) ticketing organizations claim a large share from the actual ticket price, so that the event organizers and the content creators are left with much less profit. Hence, a substitute solution without ticket organizations that leads to less profit deduction is sought. (b) Ticket scalping in high-demand events do not only lead to overpriced tickets for guests, but also, can negatively affect the event venues; Considering a scenario where a ticket scalper is able to buy 100 tickets for e.g., 50 $ each, and resell them at a price of 150 $, s/he can already make profit with only selling 34 out of those 100. In that case, if only those 34 guests instead of 100 appear at the venue and consume on-site paid services such as food and drinks Hosts will encounter a huge loss, have a high interest in eliminating ticket scalpers.
(iii) Content Creators: are producers and performers of content, such as musicians and sport teams. Their main goal is to earn the maximum amount of money by performing. However, as described above, if a scalper cannot sell all his/her tickets, the content creators having to perform for a not fully filled venue, even if the venue tickets sold out. This can reduce the energy of the performance and demotivate the content creators.
(iv) Ticketing Organizations: are the third party entities who generate and distribute the tickets and enable the ticket purchase for users. In some cases, even Hosts are also in handling the ticketing locally using a ticketing system.
(v) Affiliates: are the collaborating organizations which play an intermediary role between the Guests and the Hosts. Affiliates are marketing companies or influential people that advertise the venue to the event guests. For instance, a university can be an affiliate for an event. In this case, the university may have a special authority in distribution of tickets, or it can offer a discount to students.

Interactions of these entities within ticketing systems demand a systematic design, which not only overcome the black market concerns experienced in centralized ticketing systems, but also consider requirements of each entity involved.

### Existing Ticketing Systems

The investigation of the most relevant BC-based ticketing management systems reveals the following one: *Origin Protocol* [6], *GUTS* [7], *Blockparty* [8], *Aventus* protocol [9], and *Blocktix* [10]. A few other less related ticketing solutions exist such as [11] which explores the potential of Non-fungible tokens of Ethereum in identifying tickets or the work of [12] that is based on a BC called Consensus. Finally [13] proposes a mobile ticketing system exploring the potential of multi-signature features offered by SCs of Ethereum. Table 1 covers a comparative overview of the studied related work.

**Table 1:** Overview of the BC-based ticketing systems (N/A: not available) [19]

	Blocktix [10]	GUTS [7]	Aventus	Blockparty [8]	Origin protocol [6]	DeTi
Native token	TIX	GET	AVT	BOXX	OGN	✓
Form of linked identity	✗	✓	N/A	✓	Social profiles	Social trust, Local and external KYC
Reselling possibility	✓	✓	✓	✓	✓	✓
Mechanism for pre-sale	✓	✗	✗	✗	✗	Time-independent lottery
Internal currency	ETH	ETH, GET, Fiat	ETH, AVT	Fiat, BOXX	ETH, DAI, USDT, OGN	ETH
Guest payment currency	ETH	Fiat	ETH	Fiat	ETH, DAI, USDT	ETH
Stablecoin integration	✗	✗	✗	✗	✓	✗
Fiat currency gateway	✗	✗	✗	✗	✗	✗
Fiat gateway provider	✗	N/A	✗	✗	✗	✗
Event spam prevention	✓	✗	✓	✗	✗	✓
Can tickets be sold for less values?	✓	✗	✓/✗	✓	✓	✓
Can tickets be sold for higher values?	✓	✗	✓/✗	✓	✓	✓
Multiple tickets on account?	✓	✓	✓	✓	✓	✓
Metadata storage	IPFS	Own server	N/A	IPFS	IPFS	IPFS
Escrow account	✗	N/A	N/A	✗	✓	✗
Provide a mobile app?	✓	✓	✗	✓	✓	✗
Provide a web app?	✓	✓	✗	✓	✓	✓
User controls private key?	✓	✗	✓	✗	✓	✓
Who pays for TX fees?	User	User	User	User	Platform, User	User
Affiliate marketing	✗	N/A	✓	✓	✗	✓
Fraud prevention?	✓	✓	✓	✓	reviews, Social profiles	Social Trust, ID Approvers
Deposit for hosting an event?	✓	✓	✓	✓	✓	✗
Off-chain ticket validation	✗	✓	N/A	N/A	✗	✗
Does it preserve privacy?	✗	✗	✓	✓	✗	✓
Fee structure	Paid promos	N/A	N/A	N/A	Paid promos	Determined by Event Host
Second layer for scalability?	✗	✗	✓	✗	✗	✗

Our studies show that IPFS is the most favored Distributed Storage System (DSS) used by these work; IPFS is a Peer-to-peer (P2P) public DSS for storing and accessing data objects, files, websites, and applications. IPFS utilizes content addressing, i.e., assigning addresses and fetching files according to data object content —hash of the content—, instead of its location in the distributed storage network [14]. Whereas, Ethereum is used by most of the ticketing systems studied as their underlying BC infrastructure. A set of existing concerns with the studies related work is discussed as follows.

*Origin Protocol* [6] is not streamlined for event ticketing, since their focus mainly is put on decentralized marketplaces and e-commerce. Therefore, managing events and issuing tickets cannot be done within their application. However, their implemented architecture promises a decentralized solution to the digital asset identity management problem.

*GUTS* [7] and *Blockparty* [8] do not support true ownership by letting the users manage their private key. Although these systems market their application as decentralized ticketing platforms, users’ private keys are managed by these companies. This makes the these companies a central point of failure and puts all the tickets in the system at risk.

Except for the *Aventus* protocol [9], a second layer solution—a second layer solution refers to any processes handled outside the BC employed enabling P2P communications in an off-chain manner for higher TX throughput— has not been implemented for any of the other systems. For instance, the Aventus protocol is built on top of Ethereum and handles selected TXs off-chain to overcome the scalability problem of Ethereum, since Ethereum in its Proof-of-Work (PoW) version handles only roughly 15 TXs per second, which in comparison to a centralized payment system such as Visa with over 20,000 TXs per second is considered slow. However, Aventus still benefits from Ethereum’s security and independence. TXs within the Aventus protocol are pooled into a Merkle tree, whereas the root is published onto Ethereum for security and validation. This way TXs can be processed and verified more efficiently by Aventus nodes and batched together into Aventus blocks and sent to the Ethereum BC, where they are made public. The reason for this design decision may come from the novelty of second layer solutions or due to the fact that it requires a TX on the main network to deposit funds in the second layer. And since most guests only create one TX when buying the ticket, the benefits of a second layer do not affect the majority of the event guests.

In systems such as *Blocktix* a native token, has been used for payments. In this case, event guests must first exchange their generic purpose tokens, like Bitcoin or ETH (cryptocurrency of Ethereum), to Blocktix-specific tokens. Thus, this feature adds a layer of complexity to the entire application, since most users do not own such platform-specific tokens. For payments and deposits it is more user-friendly, when a common cryptocurrency or a wildly adopted stablecoin, such as DAI, can be used. However, except for the *Origin Protocol*, none of these ticketing systems investigated integrate stablecoins as a means of payment. This exposes the event host and guests to the price fluctuation of cryptocurrencies and may discourage end users from using the platform.

Except for *GUTS*, none of these systems prohibit a ticket holder from reselling a ticket for more than the original price. This inefficiency often leads to frustration for event guests and is identified as one of the main problems in the ticketing industry (*cf. *Sect. 2.3).

### Security Risks and Requirements in Current Blockchain-Based Ticketing Systems

Different ticketing systems have proposed different management mechanisms, fully or partially based on BCs and mostly using Ethereum-based decentralized systems using SCs. Thus, the key deficits experienced in these systems are discussed as follows, since potential risks do make the ticketing service provisioning a highly complex case and such risks need to be prevented.

#### Front Running

Every TX on a public BC like Ethereum is visible to everybody in the network. However, TXs are only considered finalized when they are accepted by a miner and included in a block. The time of publishing a TX and time of finalization depends on the amount of TX fees a BC user is willing to pay. If the network of BC users (i.e., the guests of ticketing systems) is clogged with unfinalized TXs, it is possible to get a TX validated with higher TX fees because miners want to maximize their profit and generally include the TXs that offer the highest fees. This leads to the “front running” problem that one TX can be finalized before some other TX that was published beforehand.

#### Random Number Manipulation

Since the distribution of tickets has to be fair, ticketing service providers need to use a random number as a seed for the lottery of the tickets. This random number has to be the same for all the nodes in the BC. One way of achieving such a random number generation, is the usage of the hash of a block, n-blocks after the current block in the chain. This approach introduces a problem, that a miner may not publish the block, whenever the random number is not in his/her favor. Such a risk could be mitigated by ensuring the reward for mining the block is higher than the reward of cheating the lottery.

#### Ticket Invalidation

When a guest enters the event with a valid ticket, this ticket has to be marked as used, in order to prevent it from being used multiple times. To change the state of a ticket on a BC, a TX has to be sent and this TX is put in a queue (pool of open TXs) to be mined and validated. This process takes some time, depending on how much gas (an amount every Ethereum BC client needs to pay miners for validating their TXs) is paid for mining this TX . During this time, it is possible for a guest that has just entered the event to offer his ticket for sale and attach a relatively high gas fee to the TX . This high gas fee would then lead to the ticket being transferred very fast. While the ticket invalidation TX, that marks the ticket as used, is not yet mined. In such a case, the new owner of this ticket can enter the event and the ticket would have been used twice.

#### Identity Fraud

An event host may want to provide guests a fair and neat ticket buying experience for high-demand events. To pursue this goal, a buyer could be asked to provide his/her identity. This step may prevent bulk-buying bots, but if this form of identity is only weakly linked to a human, such as a mobile phone number, scalpers could still buy cheap prepaid sim cards, to buy multiple tickets linked to these sim cards and sell those tickets on a black market.

#### Fraudulent Events

Any ticketing system needs to control mechanisms in order to prevent the creation of fraudulent events. This can be achieved by binding a creation deposit to an SC, i.e., ensuring that entities cannot profit from creating false events. The amount needed for deposit would be challenging to define, as it should be high enough to punish the creation of a fraudulent event but not limiting event creators with limited assets. In order to verify the legitimacy of an event, measurements such as the number of redeemed tickets could be used. Another option would be to incorporate an SC that lets the customers file a complaint, stating that the event has not been hosted at all or has been hosted but has not fulfilled its premises. A different approach to this problem would be to verify legitimate event creators, giving the permission to host events and distribute tickets only to verified entities. This approach could be prone to human bias if done manually or bring new challenges to identity verification if done through an automated service, as proposed later on for user identity verification in this work.

#### Event Spamming

The problem of event spamming may arise when any entity has the ability to create events and sell tickets without any fear of consequences. Event spamming may occur with the goal of profiting from unknowing customers or out of bad will against the ticketing system, diluting their event pool with meaningless events. Both solutions proposed in 2.3.5 could also be applied here. An additional approach to prevent this phenomenon would be to propose a minimum distance in time between the creation of two events.

#### Black Market Attack

This attack attempts to sell a ticket on a different marketplace platform such as Ebay for a higher price but still transfer the ownership of the ticket through the related SC. This attack can be achieved in the context of open and off-chain order-book decentralized exchanges, and if and only if the platform allows setting an arbitrary price e.g., below the original price on the aftermarket.

#### Location-based Addressing for Metadata Storage

Storing data on a public BC is expensive, since every node storing data has to pay for the respective volume. Thus, many decentralized applications only store hashes of files on the BC, while storing the actual meta-/data somewhere else, i.e., off chain. With this design, it is possible to check, whether an actual file stored off chain was modified, because any change in the file would result in a different hash. However, if the actual file is lost, data cannot be reconstructed from the hash and is permanently lost. Thus, redundancy and availability play important roles, when designing a decentralized ticketing system.

Thus, in a ticketing system which is based on SCs, it is desired to only store the information on-chain which is relevant to the functionality of the SC. Metadata such as event details that are not relevant for any of the methods in the SC must not be stored on-chain. However, storing a metadata file on a server is not an ideal solution, since the location of the server can change or may no longer exist. Even if the files were replicated on different machines, the nodes in the network do not know where the new location of files is, if the SC does not keep a list of all the those servers. Thus, location-based addressing for metadata is therefore prone to censorship and loss of data.

To solve location-based addressing, DSSes such as the Interplanetary File System (IPFS) could be used. IPFS employs content-based addressing, in which the storage location of an object is calculated based on its content and stored accordingly by distributed storage nodes [14].

#### Lottery Manipulation

In order to make a fair presale of event tickets, every user wanting a ticket for a certain event should have the same probability to receive a ticket. One way of addressing this need is to issue a presale, where everybody can register their willingness to buy a ticket during a fixed time frame. Instead of the first-come first-served approach, it would then be possible to have a lottery, where everybody gets a ticket with the probability defined by the number of tickets available divided by the amount of requests for the desired ticket. However, this trivial approach only works, when every user is only buying one ticket. Most of the time, it is desired by the users to be able to buy tickets for a certain group (Friends, Family, Coworkers, *etc.*). When this feature should be implemented, it is no longer trivial to calculate the probability a buyer should get his desired amount of *n* tickets. The probability of being able to buy a certain amount of *n* tickets has to be calculated in a way, that the lottery still is fair. It is crucial to not be able to maximize the probability of getting a ticket by grouping up and create multiple request of buying *n* tickets.

## Design

**Fig. 2:**
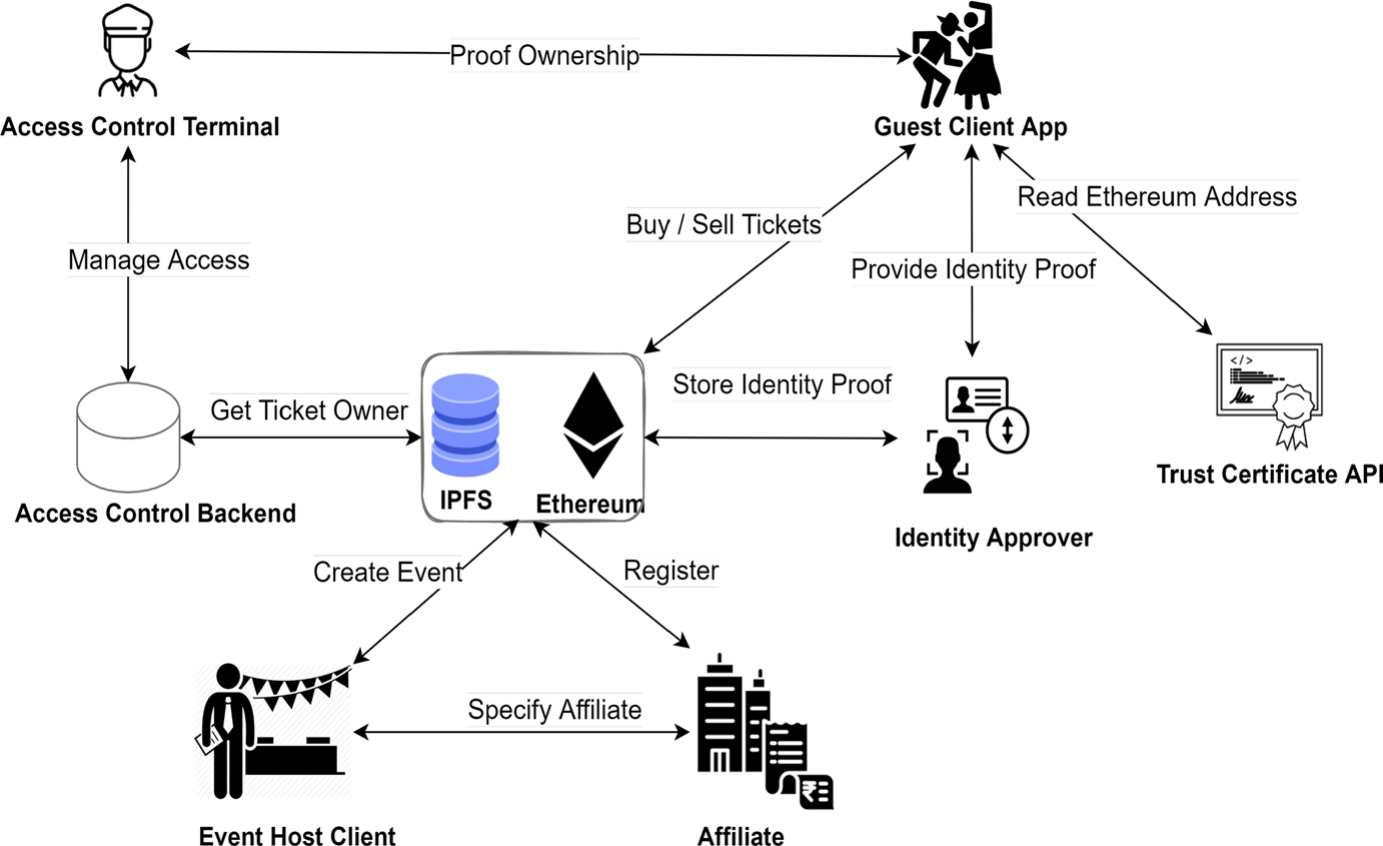
Overview of main entities and interactions in DeTi [19]

DeTi design is based fully on Smart Contracts (SC) to address the identified problems of BC-based ticketing management throughout the presale, sale, and aftermarket phases. Figure 2 shows the main stakeholders in DeTi including the event guests, hosts, ID approvers, promoters/affiliates, and Graphical User Interface (GUI) builders.

DeTi archives a trustable and transparent management of ticketing processes by decentralization of processes between the entities involved in (*i*) New event creation, (*ii*) Generating different ticket types (e.g., non-/fungible), (*iii*) Registration of guests and affiliates, (*iv*) Identity management, (*v*) Distribution of tickets, (*vi*) Access control, and (*vii*) Distribution of funds via P2P payments.

Since *gas* prices have risen by a factor of ten since April 2020 to September 2021 [15], building a second layer solution was investigated to lower TX fees. However, all layer two solutions in the Ethereum ecosystem, such as Plasma [16], require the user to first deposit cryptocurrency. This deposit TX is made on the main chain and, thus, *gas* costs add up to a similar price as directly buying a ticket from the event SC. Since these coins are locked, users cannot use them and would not be willing to deposit more than needed to buy tickets for one event. A second layer solution is sensible, if tickets are invalidated on-chain or the ownership of a ticket is transferred multiple times. Otherwise, there is no benefit for this application using a second layer solution.

To mitigate fraudulent events, it was initially intended that an event host has to make a deposit when creating an event. If many of the event guests signal that the event is fake, the deposit could be used to compensate for the fake tickets. However, this design was abandoned and instead, a more user-friendly architecture with less user interactions is in place. Trust between the host and guest can be established with social proofs and trust certificate. This design does not require any on-chain interactions to verify that the event host is who he claims to be. Furthermore, an event host often has to pay bills for the event in advance and an additional deposit that is locked until the end of an event can become a problem for event hosts.

**Fig. 3:**
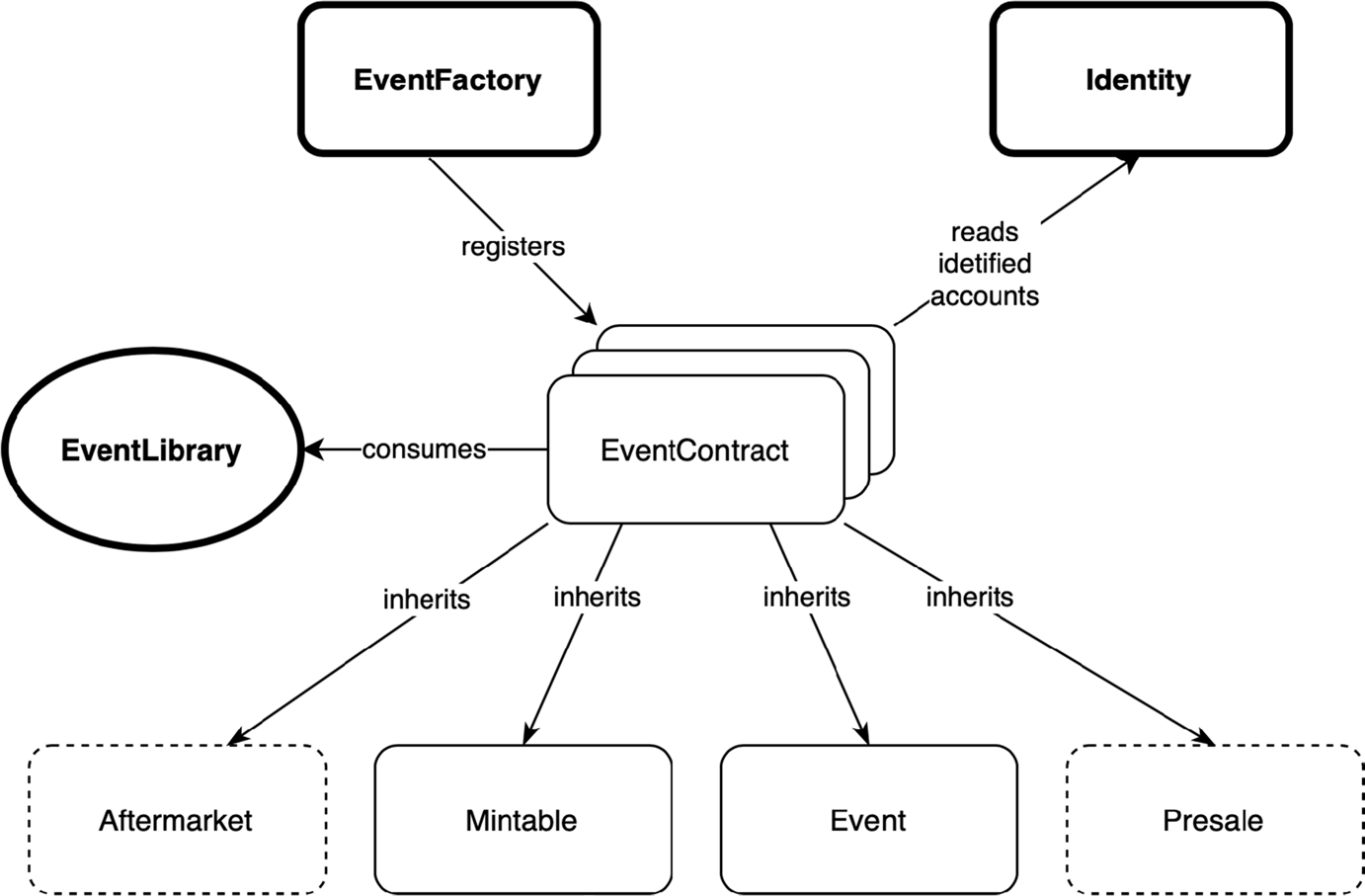
SC dependencies overview [19]

Figure 3 illustrates these SCs and their dependencies. The three bold SCs are only deployed once (when DeTi is deployed on Ethereum BC for the first time), whereas, the *EventContract* is deployed for every new event creation. The *EventLibrary* contains the common stateless functionality across multiple SCs. The SCs with a dotted outline add optional functionalities. This design pattern is chosen to reduce the deployment costs, allowing an event host to only deploy necessary functionality. For example, it is possible to create an event without an aftermarket if the host desires to do so.

### Creating an Event

**Fig. 4:**
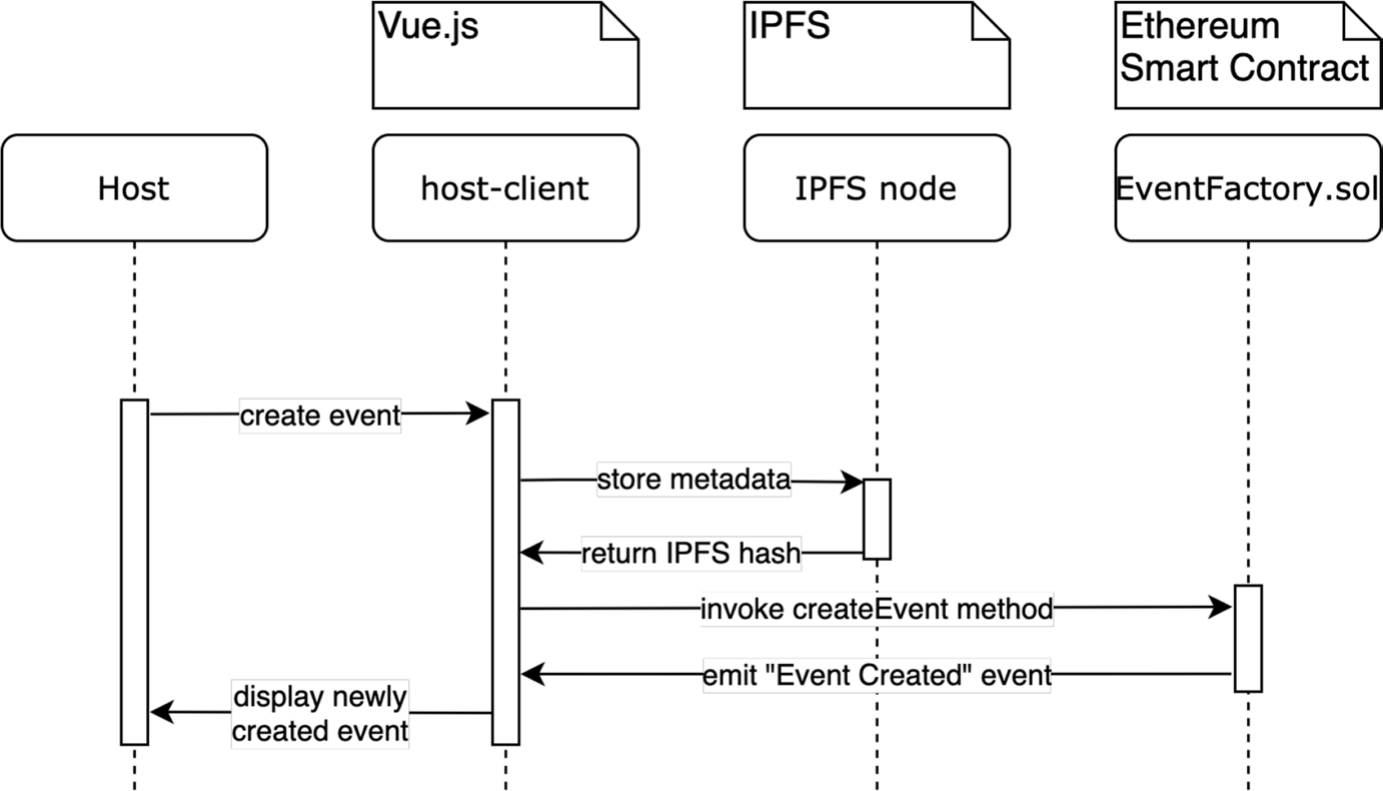
Event creation in DeTi [19]

An event host in DeTi creates an event as shown in Fig. 4. The metadata information about the event is first stored on IPFS. IPFS generates a hash from this data, which acts as an address and can then be used by anyone with a connection to the IPFS network to retrieve this data. Then, the *Event Factory* SC can be invoked to create a new event contract. This invocation requires multiple parameters, including the aforementioned IPFS hash that connects the event to its metadata. These processes are all done in an automated and decentralized fashion. The *EventContract* contains all the core functions and data structures that are used by the other SCs. DeTi builds a single SC for each event that manages the distribution of tickets and aftermarket of non-/fungible tickets. The heart of this SC is the state that keeps track of the ownership of the tickets. Therefore, it is used to efficiently store and retrieve tickets.

### Generating Tickets

The *Mintable* SC is dedicated to issuing new tickets. In DeTi, to differentiate between multiple tickets, ticket ID is built in a specific way consisting of ticket ID to indicate whether the ticket is fungible or non-fungible, the ticket type, and the ID assigned to a Fungible tokens’ ID. The Minting SC stores different ticket types using this encoding schema. Using efficient shift operations and bitwise comparators, the Ticket_ID can be decoded.

$$\begin{aligned} uint256\ Ticket_{ID} = \underbrace{1}_\text {nf flag (1 bit)} \underbrace{000...100}_\text {type (127 bit)} \underbrace{000...011}_\text {index (128 bit)} \end{aligned}$$

### Identity Verification

In DeTi a decentralized design of identity (ID) verification is made possible for ticketing ecosystems. In DeTi ID is obtained from each user through a KYC process. Since in Switzerland Sim cards (i.e., phone numbers) are only obtained after identification processes, therefore, guests can be identified by checking, their phone number ownership. Once this check is successful, the level of identity verification of the linked Ethereum address is set on the *Identity* SC.

**Fig. 5:**
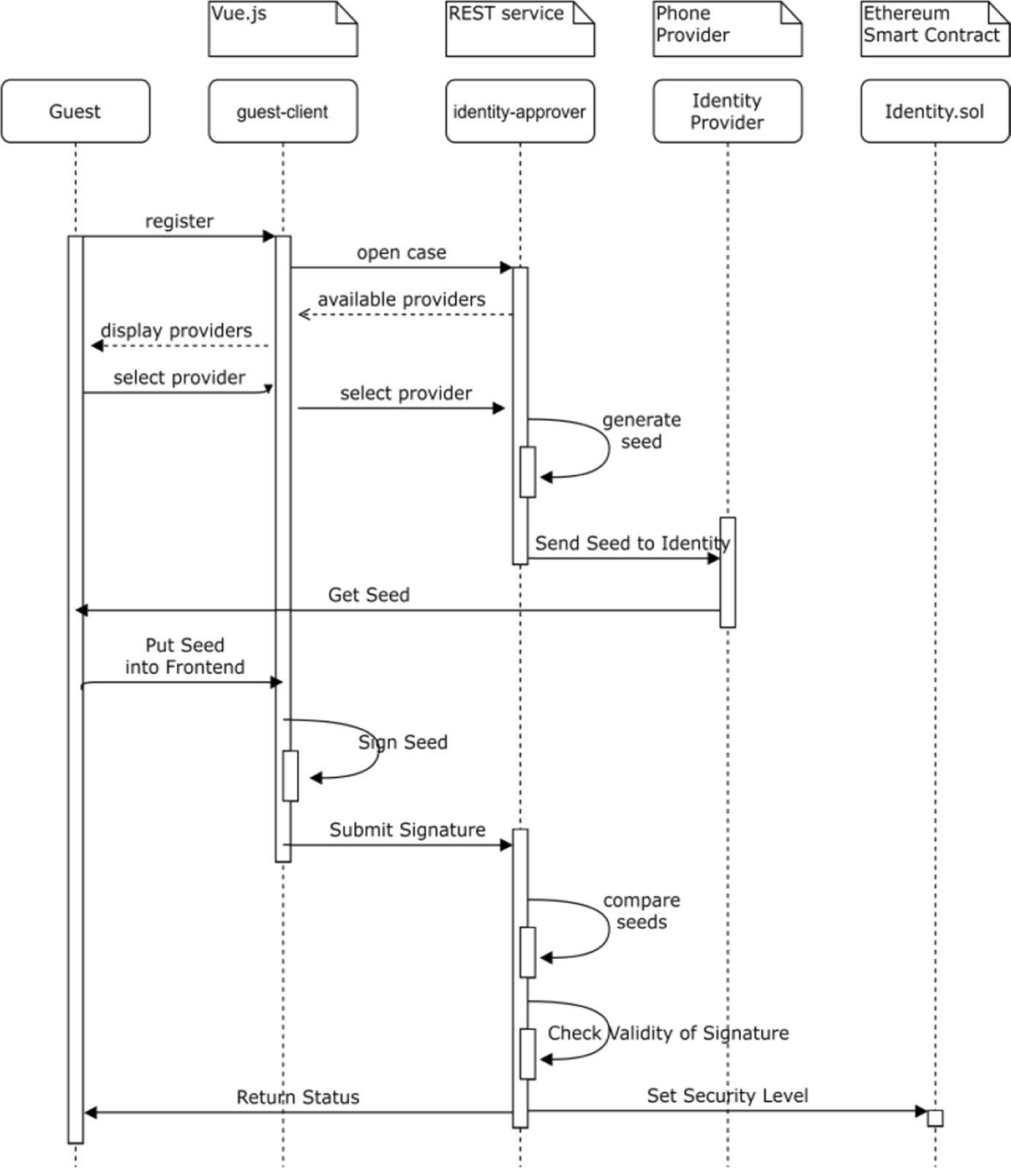
Guests identity registration in DeTi [19]

The *Identity* SC is essential to eliminate a black market as it stores identity proofs in the BC, while an off-chain service (offered by ID-approvers) checks the guest’s identity. In the case of using phone numbers, the SIM card provider company gets involved in the processes of ID verification as shown by Fig. 5. Using the phone number-based identification of users eliminates the need for more traditional methods such as presenting physical identity documents for matching a ticket bought to a ticket owner. Moreover, it is not needed to re-identify if users change their phones, but the same SIM card is being used. In case a user changes his/her phone number, he/she will need to re-apply for identification on the DeTi system to prevent the potential malicious activities.

In DeTi the *guest-client* application enables users to register an identity verification request. This request is forwarded to the ID-approver application. The proof of the identity after being verified is stored in BC, which is a link between an ID-approver’s Ethereum address and the registered (and identified) guests’ Ethereum address.

#### ID Approver

The decentralized solution for identification designed in DeTi automates the identity approving process and allows anybody to act as an ID approver. Hence, anybody may register on the identity SC as an ID approver and also use its own idea and implementation of identity proofs. In order for an ID approver to be able to save identity proofs for guests on the BC, she has to register as an ID approver first. To do this, the ID approver provides information about the kind of identity procedures she is using for ID verification. This information is stored on IPFS and the corresponding IPFS hash is saved in the SC to register the ID approver. Important to note is that the ID approver is a guest-trusted entity. However, the goal is to build an application that does not rely on a single trusted entity. Thus, in DeTi an event host can select from different ID approvers.

The ID approver is a guest-trusted entity that checks if a user is in control of a certain Ethereum address, phone number, and other identification required information. ID approver stores the unique characteristic of that ID and link it to the guest Ethereum address. As shown in Fig. 5, the ID approver generates a random sequence and sends it back to the guest. The guest receives the random sequence and enters it in the guest-client application. The guest is then asked to sign the random sequence using her Ethereum address. The ID approver checks the signature for its validity and if this check completes successfully, a proof is generated.

Via DeTi it is made possible that an event host can act as the ID approver, too. This could reduce the potential fraud by third party ID approvers. In the case DeTi is supposed to perform the ID approver role, there are three levels (approaches) of identity verification is developed in DeTi.

- The first level: verification of an email address.
- The second level: verification of a phone number. In DeTi Twilio [17] is used to send a text message to a phone number.
- The third level of identity is the verification through a KYC-like process.

For the third level, a local KYC system is developed in DeTi based on “AWS Rekognition” [18] to compute the similarity of the faces provided (e.g., a “selfie” compared to photo on ID card), which is done by uploading the image and ID files to AWS. Moreover, the “*Tesseract*” library is used to process a picture containing text. Hence, an image is processed by extracting the text of a given image, using a pre-trained Optical Character Recognition (OCR) model.

In DeTi only the *level* of proof linked to a guest’s Ethereum address needs to be stored on the BC (i.e., a digit number). Confidential data is only stored off chain by the ID-approver.

#### Social Trust Certificates

DeTi does not restrict any user from creating an event, and it mitigates fraudulent events from being created by malicious entities. A common design pattern for this problem is an on-chain voting mechanism. A mostly used approach is creating a multipurpose token for governance purposes. Token holders become eligible to cast votes if they believe an event is fraudulent. In this case, the event creators must deposit some currency when creating an event. Furthermore, they must make an on-chain TX to vote on suspicious events. This makes the User eXperience (UX) more complex for the event hosts and causes a fragmented ecosystem.

In DeTi design, the goal is to enable users to verify events legitimacy without the need of a governance mechanism. Furthermore, the expected trust level needed from third parties shall not be greater than in the current ticketing ecosystem. In this regard, the *social trust certificates* are used to enable a user to check whether an event is legitimate or not without the need of a third party. Hence, event hosts upload the same public key, that is used for creating the event, to a social profile or the official event website. This way, an event host can prove her ownership of the event, the social profile, or that website.

Event guests can retrieve the URL of the website or social profile from the SC and then verify themselves if the event is legitimate or not. However, this is not 100% foolproof. For an imposter, it is easy to fake an official Web site with a slightly different URL or social media account. However, aggregating multiple social media platforms makes it harder for an attacker and thus, increases the legitimacy of an event compared to today’s standards.

**Fig. 6:**
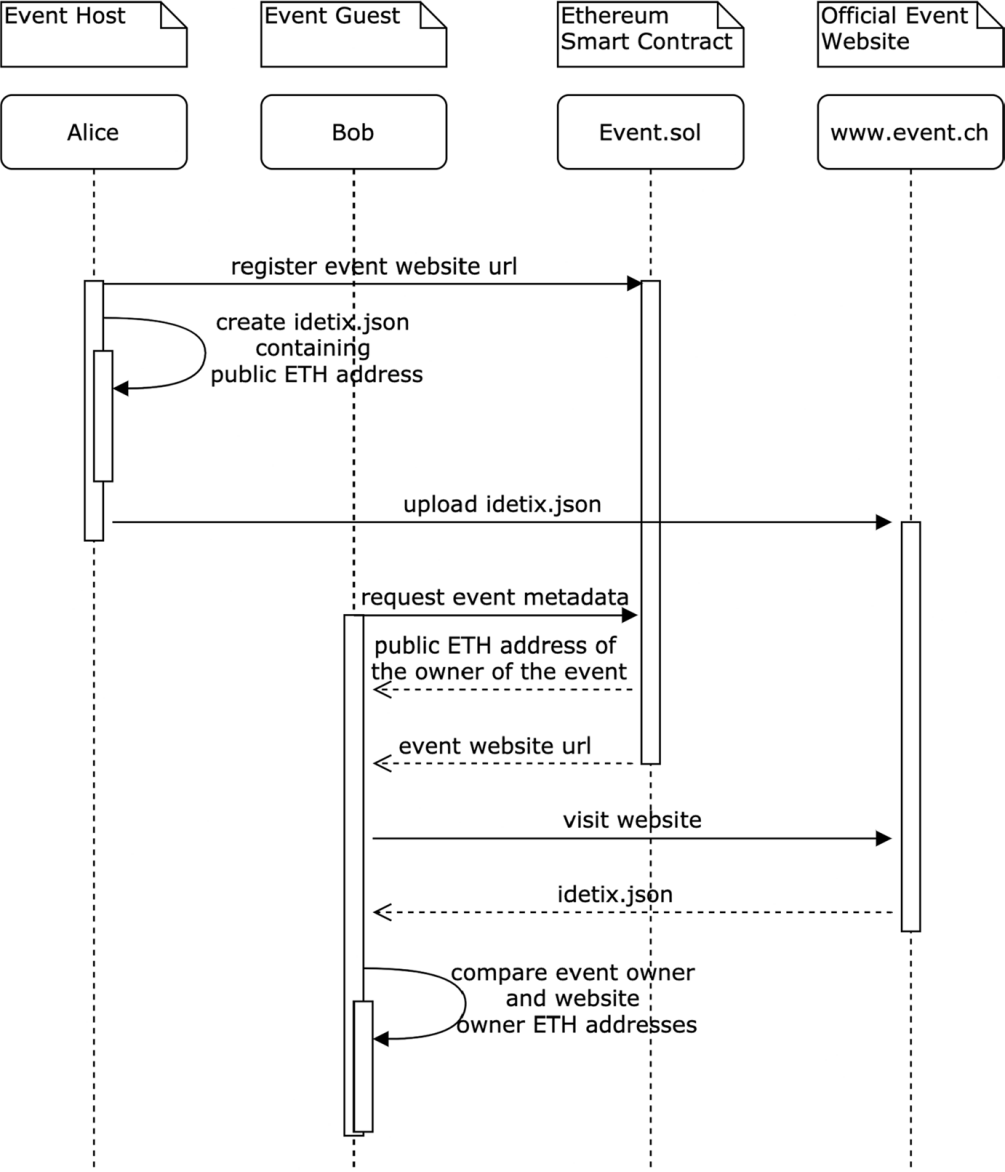
Social trust certificated on the event web site [19]

Figure 6 illustrates how an event host can use the official event website to increase the authenticity of the event in DeTi. Similarly, this procedure can be applied to any social profile such as Twitter, Facebook, and Instagram. Thus, instead of uploading a JSON file or HTML meta-tag to the website, the even host’s public Ethereum address is included in that particular website or social media platform.

### Managing Presale Processes

Whenever an event takes place and the demand for the tickets is high, potential guests will try to buy tickets when the sale period starts. This leads to high traffic on the ticket sellers (e.g., hired by an event host) website. In such a case, successful buying is often not guarantied, and it is affected by guests’ Internet bandwidth.

To prevent this problem and to have a fair and transparent experience in the initial ticket distribution for every guest, a “lottery system” is designed in DeTi. Thus, every guest that wants to attend the event can place a buy order during the presale period. This order will cost an equivalent amount to the ticket price. At the end of the presale period, the tickets will be allocated to buyers using a random number. In case a guest has not won a ticket, she reclaims the spent money. If there are more tickets than interested buyers in the presale, each of them receives a ticket.

DeTi adopts the design pattern of the *future blockhash* to generate a random number. During the creation of a presale ticket type, a future blocknumber is defined. This block acts as the closing date for the presale as well as the source of randomness. There is a chance of manipulation by the miner that adds the block with the defined number by not publishing a block if randomness is not in his favor. However, in order to do so, the miner must resign on the block reward. In a real life implementation, if this is an actual threat to a ticketing platform including DeTi, the prices have to be larger than the ticket price.

To implement fair odds for the presale lottery, a user can only place one presale order for one ticket. The presale order will be placed in a list on the BC and every new presale order will be appended to the list. When the presale is over and the lottery is triggered, a random number in the range from zero to the number of presale orders is generated. The list of buy orders will then be cast to a ring by connecting the head with the tail of the list. The winners of the lottery are defined by the *n* guests starting at the index, which is defined by the generated random number.

**Fig. 7:**
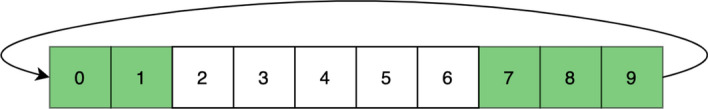
Ring lottery example [19]

Figure 7 shows an example where ten people signed up for the lottery, but only five tickets are available. The random number returns 7 which indicates the starting winner number. The green boxes indicate the winners.

**Fig. 8:**
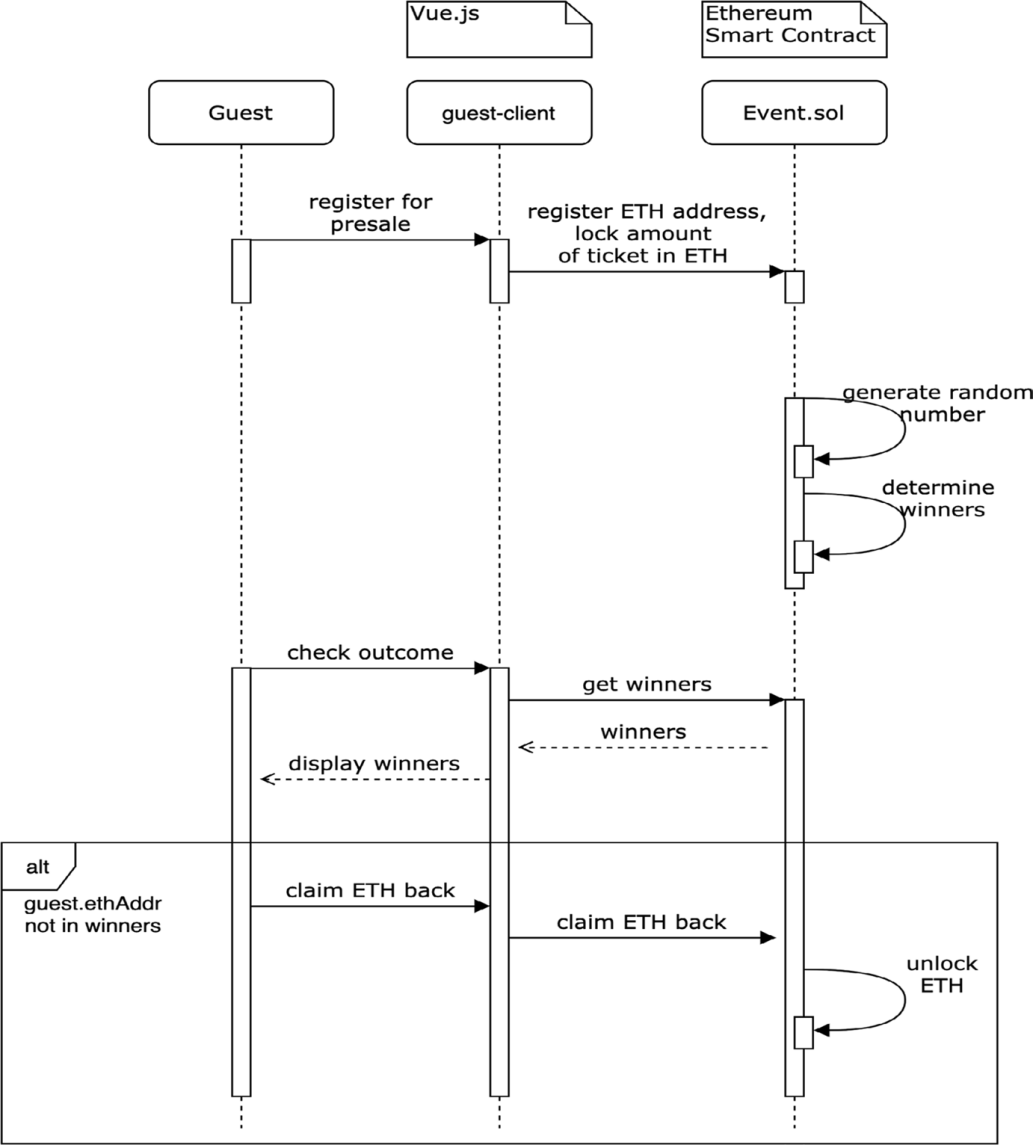
Presale sequence diagram [19]

Figure 8 illustrates how a guest can participate in a ticket presale via DeTi. When registering for the presale, the ticket price is locked in the *EventContract* SC. A user has the option to opt-out at any time and receives the locked *ETH* back. For every event, it is the event host who decides when the presale period ends. After this defined end time, the *EventContract* SC uses a source of randomness to determine the winners of the presale. If the guest is among the winners, he can mint his ticket. If not, the guest can claim her spent *ETH* back.

### Buying a Ticket

**Fig. 9:**
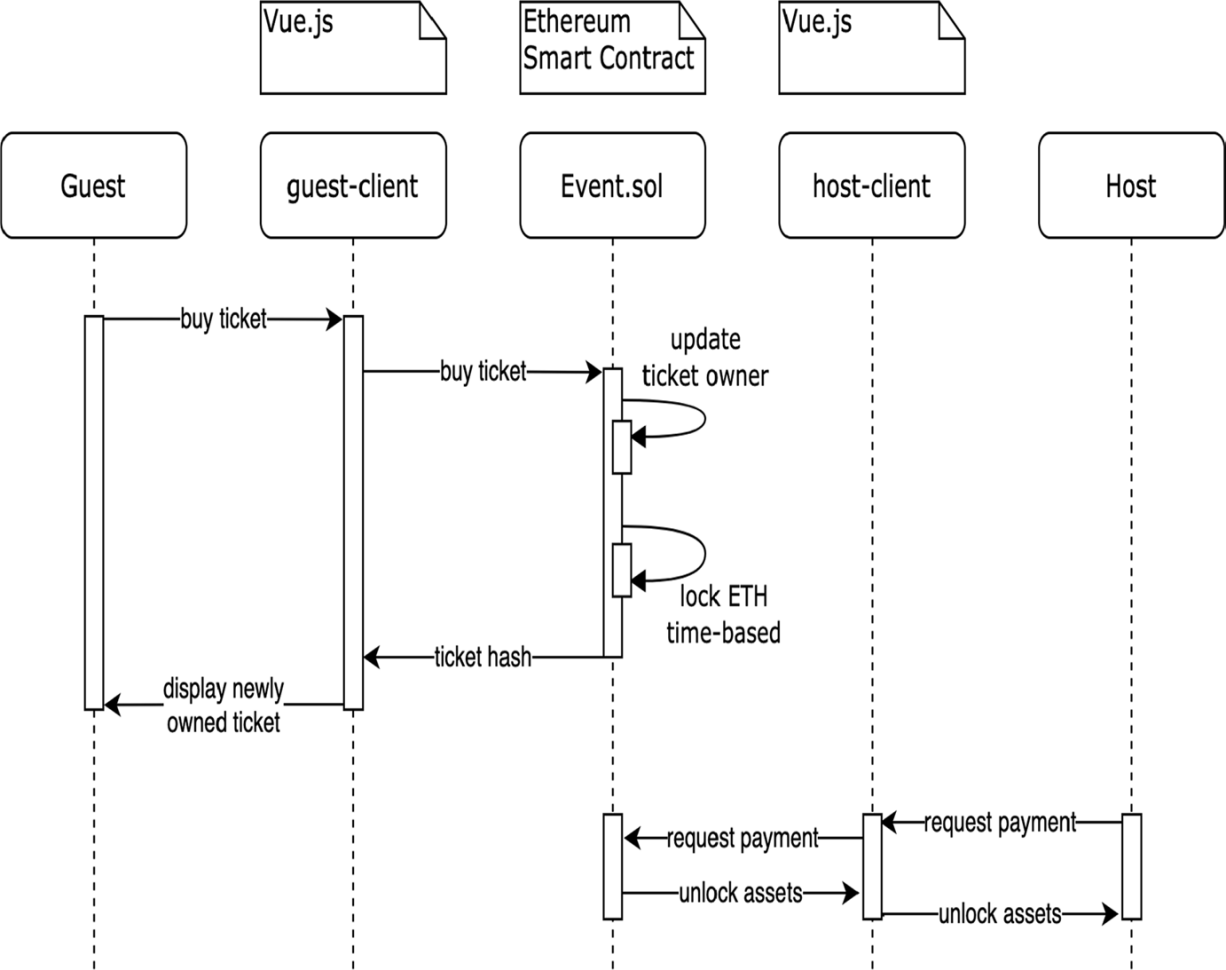
Ticket buying sequence diagram in DeTi [19]

Figure 9 shows the process of buying a ticket. A guest interacts with the guest-client application to buy a ticket. The client application then sends the requests to buy a ticket to the *Event* SC. The contract updates the owner of the bought ticket and locks the price in *ETH* until the event date has passed. After the event, the event host can request to receive the now unlocked *ETH* from *Event* SC to initiate the payment. This step is necessary since a SC cannot initiate a TX itself. In this phase also, the ticket allocation and funds transfer are being done in a decentralized fashion.

### Buying a Ticket from an Affiliate

**Fig. 10:**
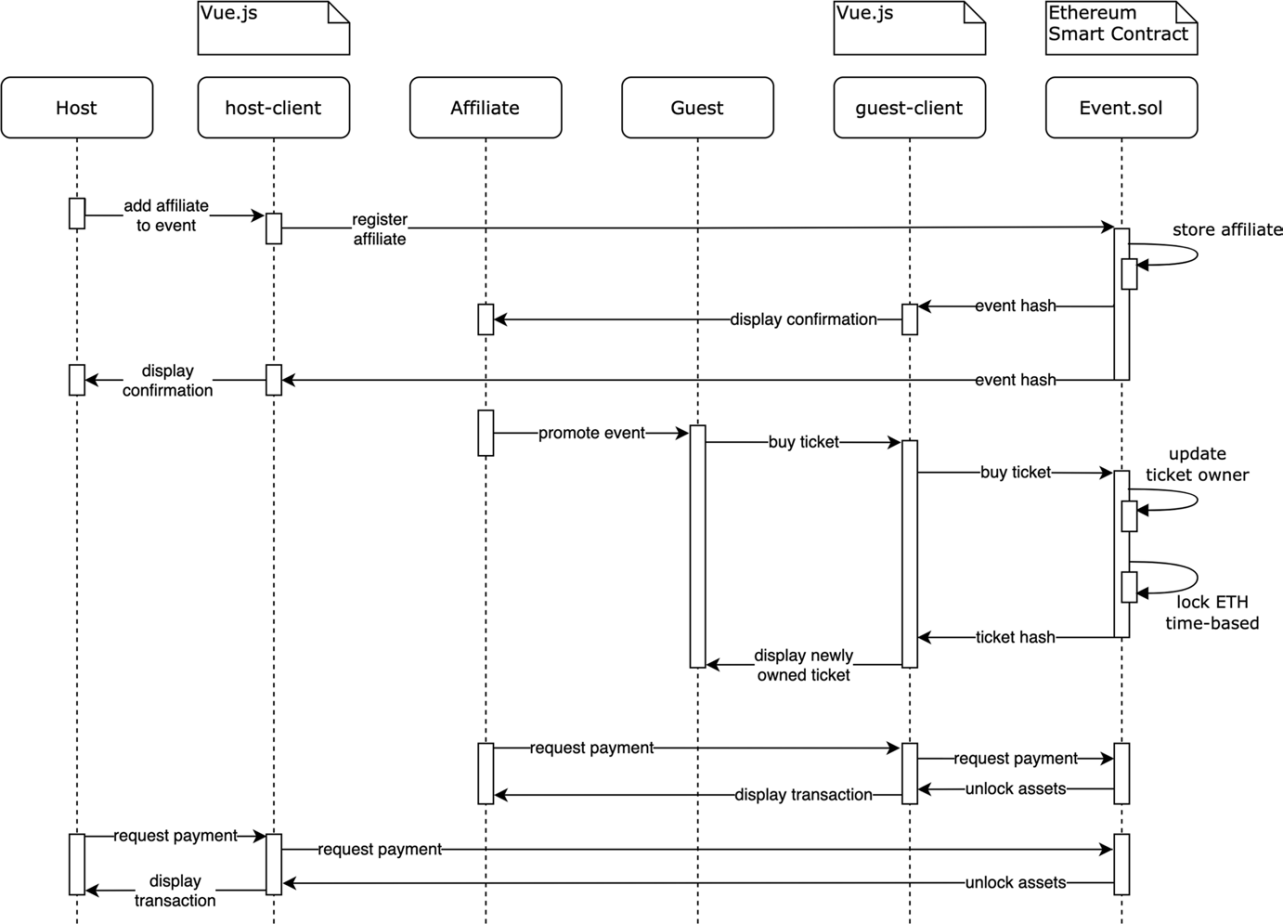
Managing ticket sale or buying a ticket from affiliate in DeTi [19]

To broaden an event’s visibility, hosts may want to contract affiliates to promote their events. Via DeTi hosts can register affiliates on the *Event* SC, such that the affiliates can distribute a link containing their Ethereum address as a query segment. The buying process is the same as in Fig. 9. However, as shown by Fig. 10 the affiliate’s address is also forwarded to the SC and is linked to the bought ticket as well. After the event, when the ticket prices in *ETH* are unlocked, the affiliate can request to receive the award from each ticket that was sold holding his address as affiliate.

### Reselling a Ticket

**Fig. 11:**
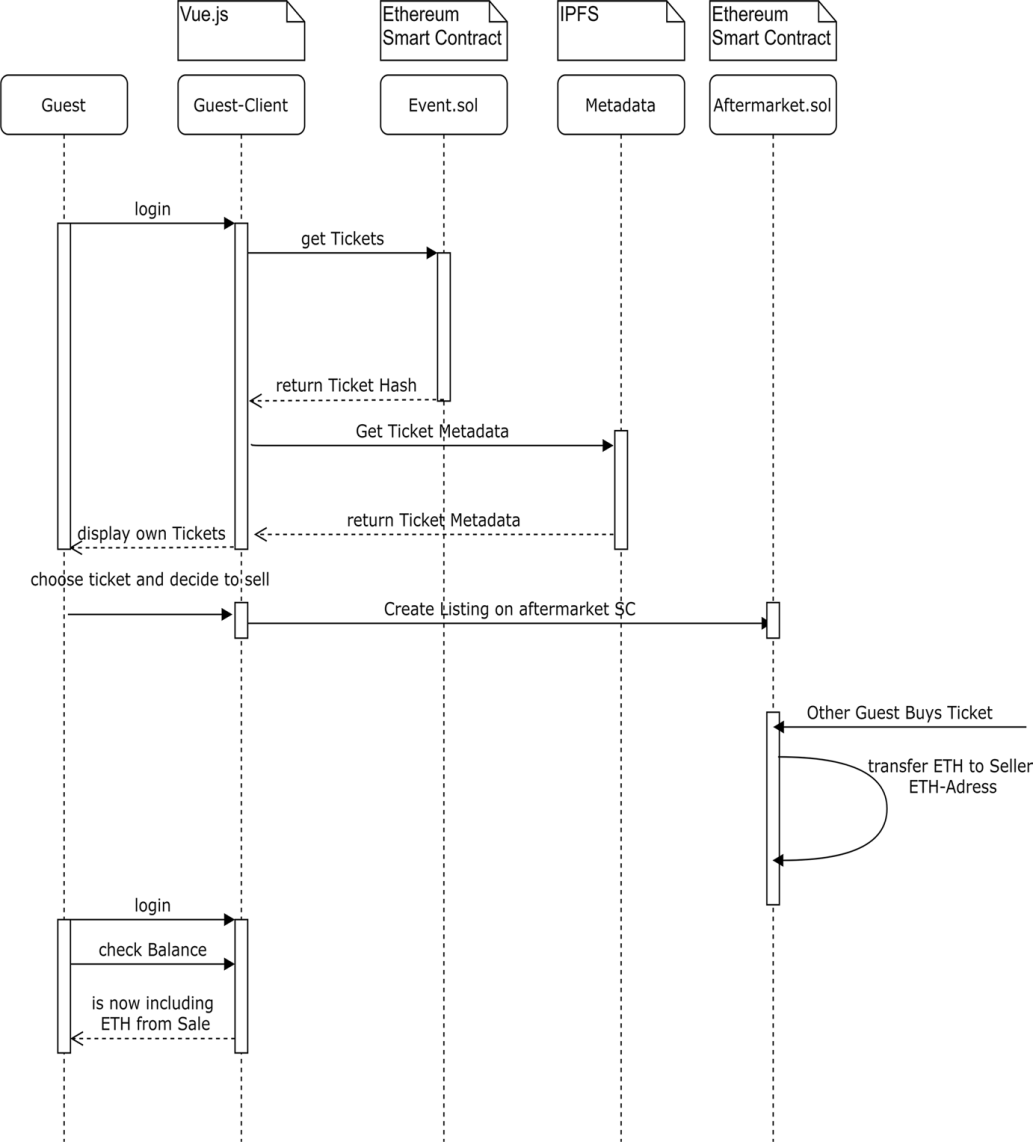
Reselling tickets in DeTi [19]

When an identified guest logs into her account, the guest-client application gets all the tickets owned by the guest that are still valid. Guest-client application automatically retrieves the ticket metadata from the IPFS storage and the guest sees her tickets. Guests can choose a ticket which no longer want to have and therefore chose to sell their ticket via the guest-client application which lists the ticket on the aftermarket. Whenever the ticket is bought by another guest, the payment is directly transferred to the seller’s wallet. This process is illustrated by Fig. 11.

### Buying a Re-sold Ticket

From the customer’s point-of-view, the process of buying a resold ticket is identical to buying directly from the event host. The Guest first connects to her wallet through her private key on the guest-client application. Then she selects the event she is interested in and chooses to buy a ticket, which invokes the corresponding *EventContract* SC. The SC locks the price of the ticket until the event has passed and returns the ticket hash to the guest-client application, where it is displayed to the guest.

### Managing Aftermarket

This optional building block materializes a decentralized exchange to DeTi fully based on SCs. One of the goals of DeTi is to build a regulated but decentralized aftermarket and to prevent the emergence of a blackmarket with unregulated prices. Thus, it must not be possible to find a seller on a different platform and perform the ownership transfer through the regulated aftermarket. With the architecture of existing decentralized exchanges, such an attack is possible.

This problem is solved in DeTi by using a queuing system for the aftermarket. The *Aftermarket* SC enforces a user to sell or buy tickets from the head of the aftermarket buy/sell queue. A ticket is always sold to the person that created a buy order first.

**Fig. 12:**
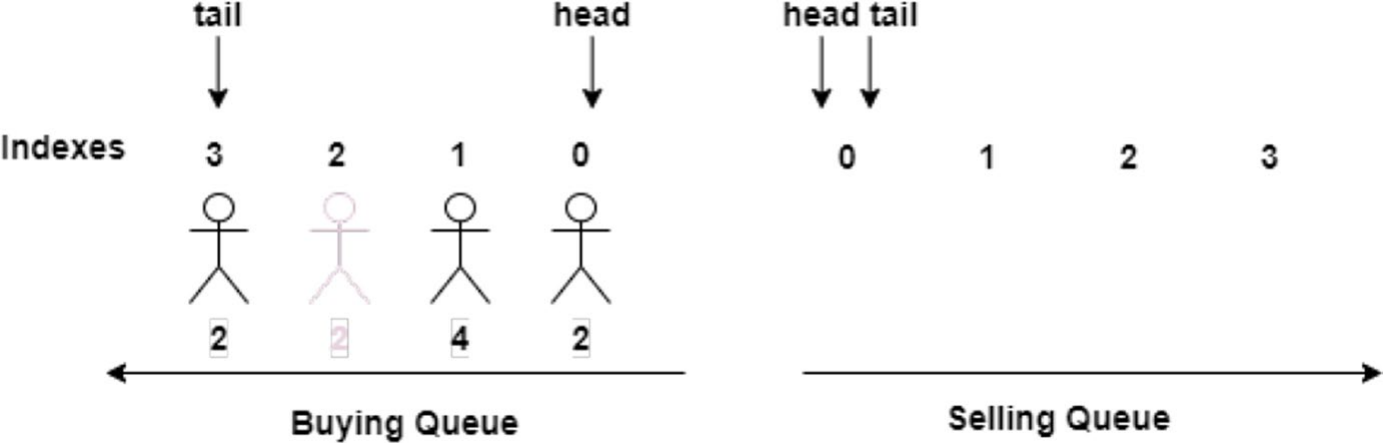
Automated aftermarket management with buy and sell queues [19]

DeTi manages the aftermarket by designing a *buying queue* and a *selling queue* where users are queued up for buying tickets or selling their previously acquired tickets, as shown in Fig. 12. DeTi automatically matches these users if the opposite queue is not empty. The number below each person indicates how many tickets she is willing to buy or sell. A *head pointer* is used in both queues to find the next buyer/seller in constant time. The *tail pointer* is used to enqueue a new buyer/seller. However, it is possible that a person leaves the queue as indicated with the potential buyer at index 2. This user is no longer interested in buying a ticket. The state management in DeTi does not allow having the same users in both queues at the same time.

#### Dynamic Pricing

DeTi allows only reselling the tickets with prices lower than the original price. This decision is made to prevent from creation of black markets. Thus, an architecture is designed to accomplish multiple buying and selling queues with fixed prices. Creator of an event (e.g., a host) can configure the aftermarket with an arbitrary number of queues.DeTi sets fixed prices by using a percentage of the original prices and the granularity (i.e., number of queues) can be configured by the event owner.

#### Aftermarket Scenarios

**Fig. 13:**
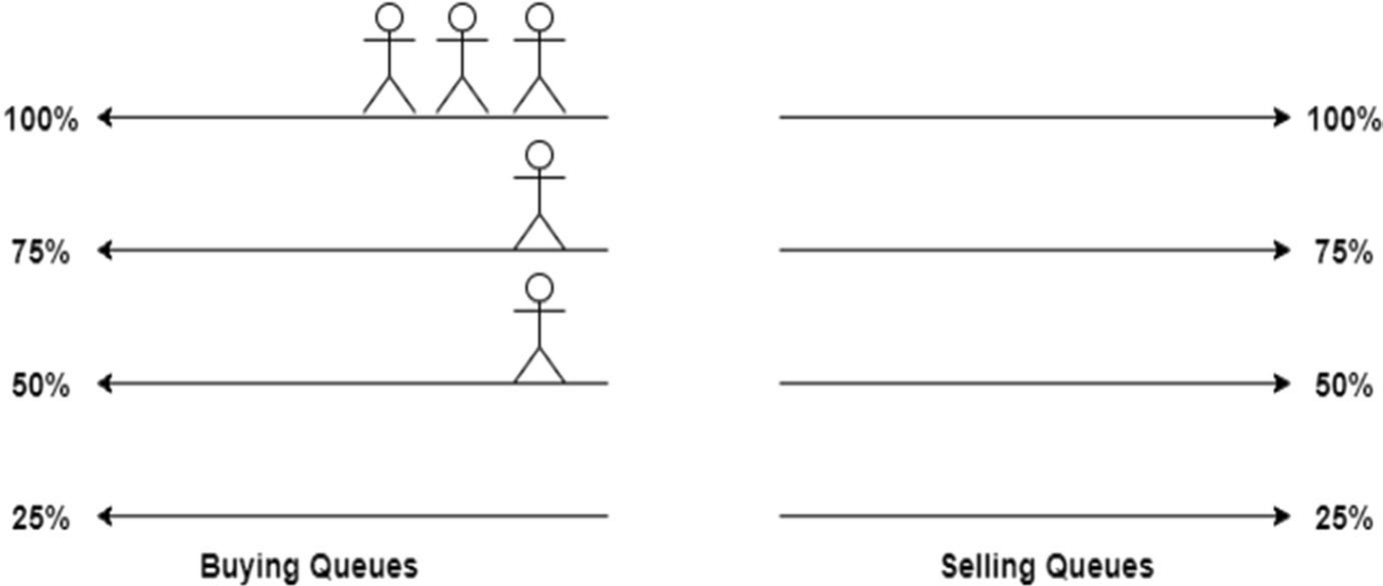
Managing aftermarket with high demand [19]

The following scenarios describe how possible states may occur of the DeTi’s aftermarket. Figure 13 shows how multiple people queued up in the buying queues, meaning that the demand for this particular type of ticket is high. A ticket owner can immediately sell her ticket to any person at the head of each queue. Of course, to maximize profit, one should always choose the queue that offers the highest price. Contrarily, the same state can occur in the selling queues. Meaning that the supply is higher than the demand. A buyer can instantly fill a sell order.

**Fig. 14:**
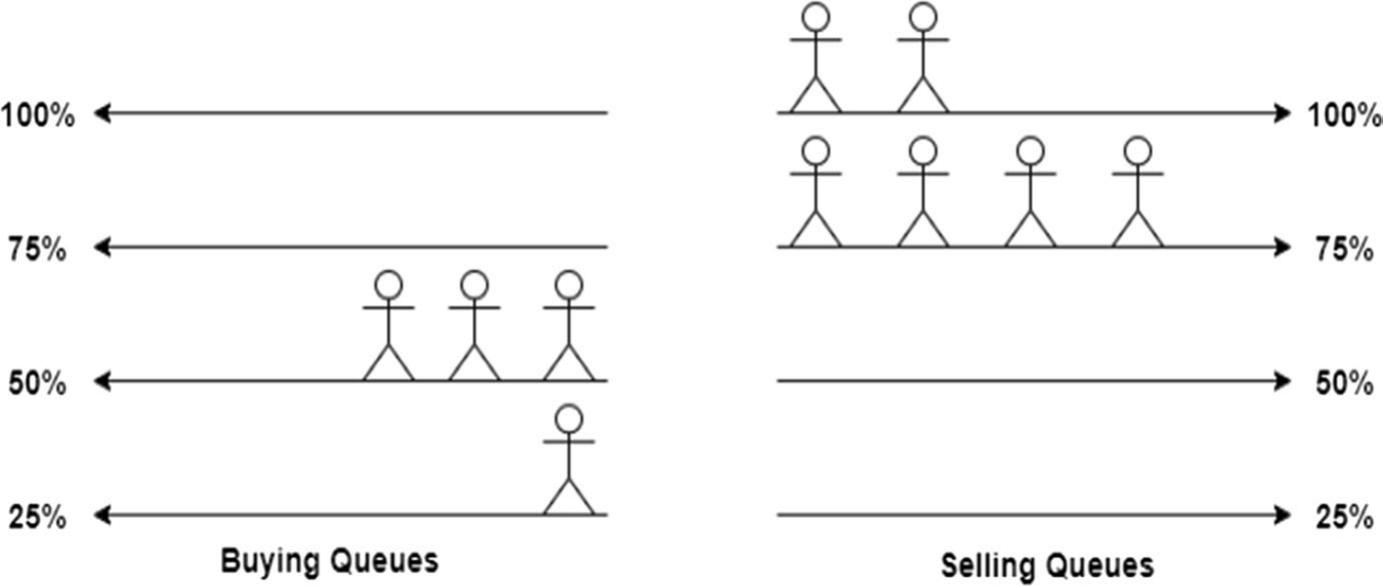
Aftermarket with lower prices than original ticket price [19]

Another possible scenario is shown in Fig. 14 where multiple people are willing to sell the ticket for 75% of the original price. On the other side, multiple people are willing to buy a ticket for only 50% of the original ticket price. In this state, any seller could immediately settle a trade at 50% of the original price and any buyer could immediately fill an offer at 75%.

**Fig. 15:**
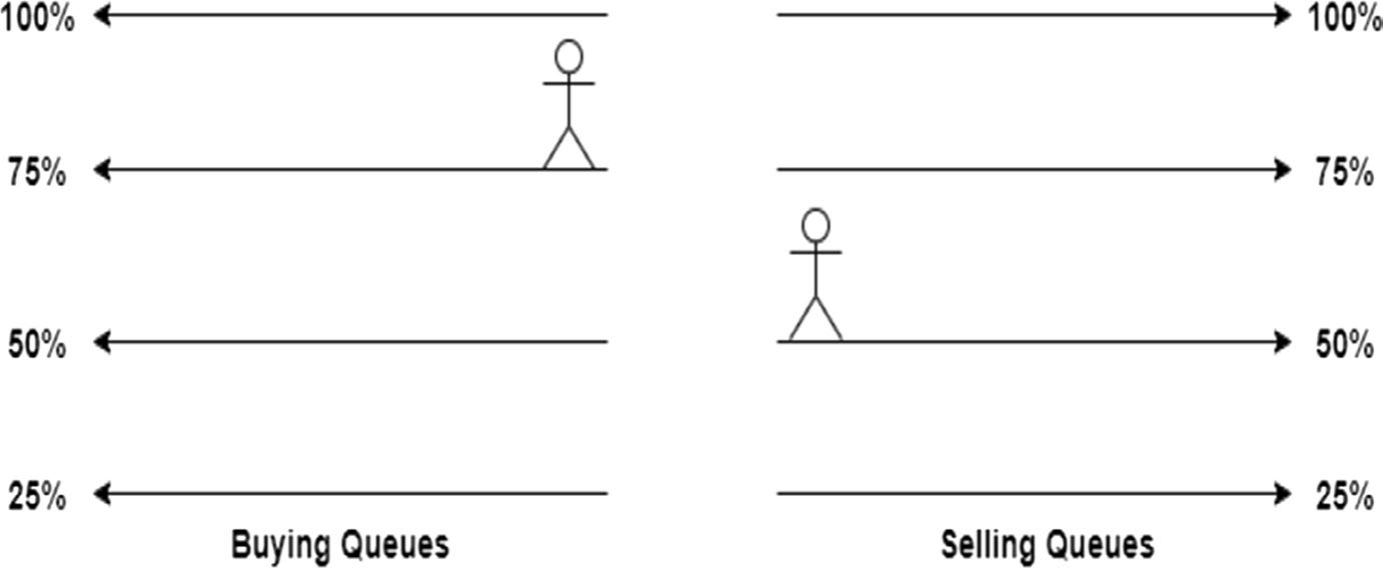
Aftermarket with arbitrage opportunities [19]

The third scenario is when people join a wrong queue. This can occur when a sell and a buy order are processed in the same block or due to wrongful interaction with the SC as shown in Fig. 15. There is a person willing to sell a ticket for less than the highest offer on the buyer side. This opens the possibility of arbitrage trading. A third person can buy the ticket at 50% and immediately resell the ticket again for 75%.

There are two solutions to solve that problem. Joining the wrong queue due to human error can be eliminated by warning the user in the frontend application that joining the selected queue results in a loss of opportunity cost.

Solving the issue in the SC comes at the price of efficiency. An additional parameter can be introduced to keep track how many people are in each queue. Before each trade, the SC evaluates if the described state occurs. However, depending on the granularity of the queues, it might not be feasible to loop over each queue as this results in higher network fees and might lead to an *out-of-gas exception*.

In DeTi, a warning in the frontend is displayed when a user attempts to join a queue that is not optimal.

### Access Control

To provide a solution for the full life-cycle of a ticket, an access control application, and an access terminal have been designed and implemented for DeTi to check a guest’s ownership of a required ticket. These two applications guarantee that only legitimate guests, that are holding a valid ticket, may enter an event. The process of invalidating tickets is done off-chain due to scalability limitations of the Ethereum BC. The off-chain invalidation requires that tickets cannot be resold after they have been used to enter the venue. Hence, a passive invalidation mechanism is implemented via the SCs making it impossible to resell a ticket after the event doors are open and guests can enter to the venue.

As the guests attempt to gain access to an event, they will be confronted with the event entrance control system. Since a guest’s ticket is linked to their Ethereum address, thus, guests just have to prove their ownership of the given Ethereum address. In order to do this, a guest has to provide a signature of a message. This signature together with the message and the guest’s public key can then be verified, which ultimately proves that the guest owns this Ethereum address.

The access terminal Web application is designed to run on tablets at the event. These tablets are used to display messages to be signed by guest-client applications. An embedded camera feature in the guest-client application is considered to scan the messages at the entrance terminals. Using the guest client application, guests sign the message. the signed message is automatically sent to the DeTi access terminal backend for verification.

**Fig. 16:**
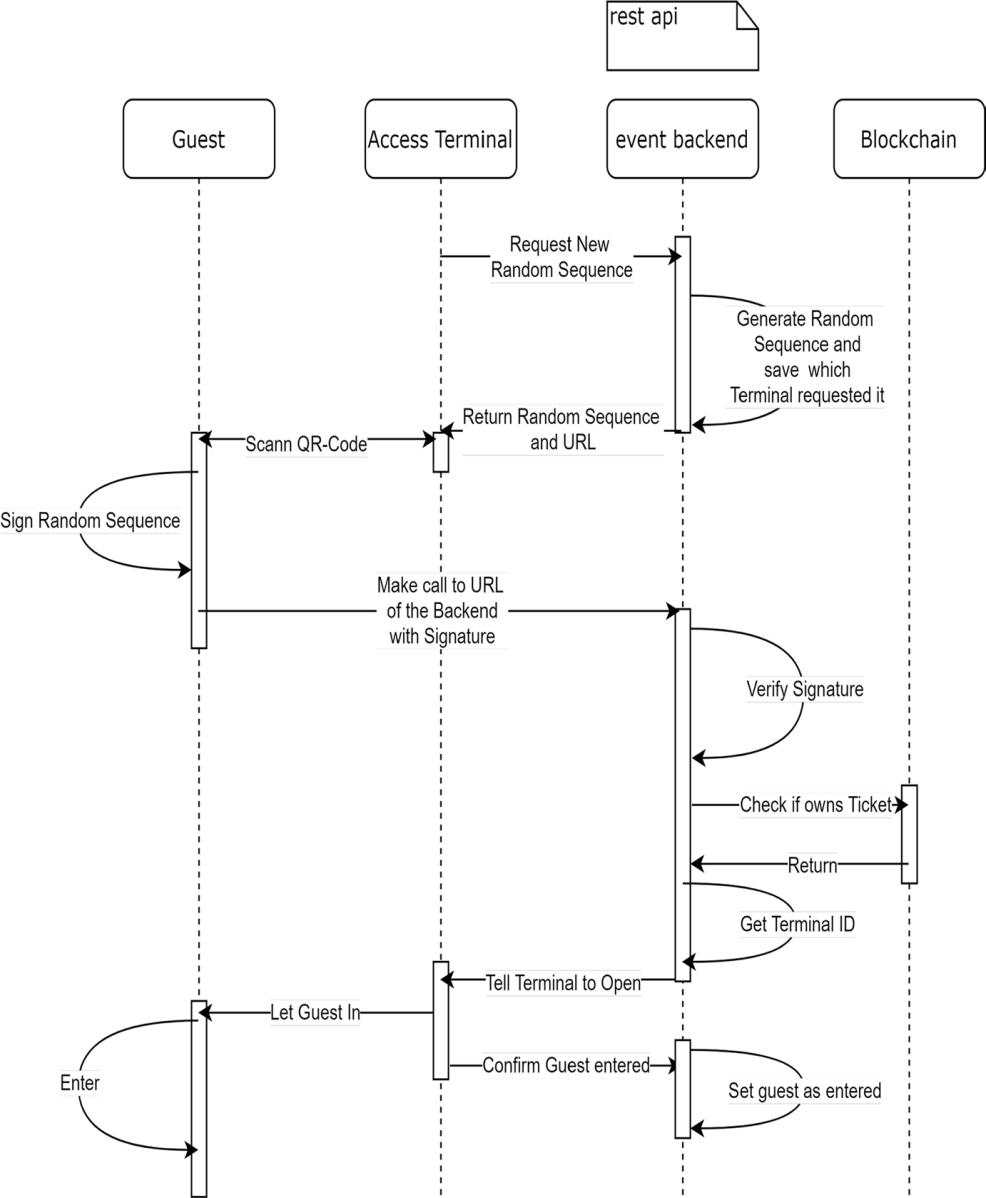
Access control in DeTi [19]

DeTi backend provides an API to register access terminals, create new messages for signing, verifying signatures, and checking whether a ticket of the guest actually exists and was not already used to enter the event. The entrance control terminals are registered with the DeTi backend. As shown in Fig. 16 every access terminal receives its unique identifier. The terminal then requests a unique random sequence, which is stored with the corresponding terminal ID in the DeTi backend. This random sequence is then displayed alongside the DeTi backend URL as a QR code on the terminal.

The guest scans the QR code and extracts the random sequence. She then proceeds to sign the sequence using her Ethereum address, proving her ownership. The signature, the Ethereum address, the number of tickets, and the random sequence are then sent to the DeTi backend. The DeTi backend then evaluates the validity of the signature. After that, the BC is queried on whether the random sequence exists and if the Ethereum address actually holds enough tickets. It is also checked whether this ticket already entered the venue. If all these checks are successful, the terminal, specified by its ID, is messaged to let the guest pass. At the same time, the Ethereum address and the corresponding ticket are added to the database, which tracks the area the ticket owners just entered. This implementation also holds for changing from different areas in a venue, for example accessing the VIP area from the general area of the venue.

The guest presents her ticket to the employee at the gate in form of a QR code on her phone. The employee scans the code using the ticket scanner mobile application, which invokes a call to the rest API on the event DeTi backend, which contains a database with all tickets and the public keys of their respective owners. Once the ticket scanner application receives the confirmation from the server that the code is valid, the guest is granted access to the event.

### DeTi’s Guest Application

**Fig. 17:**
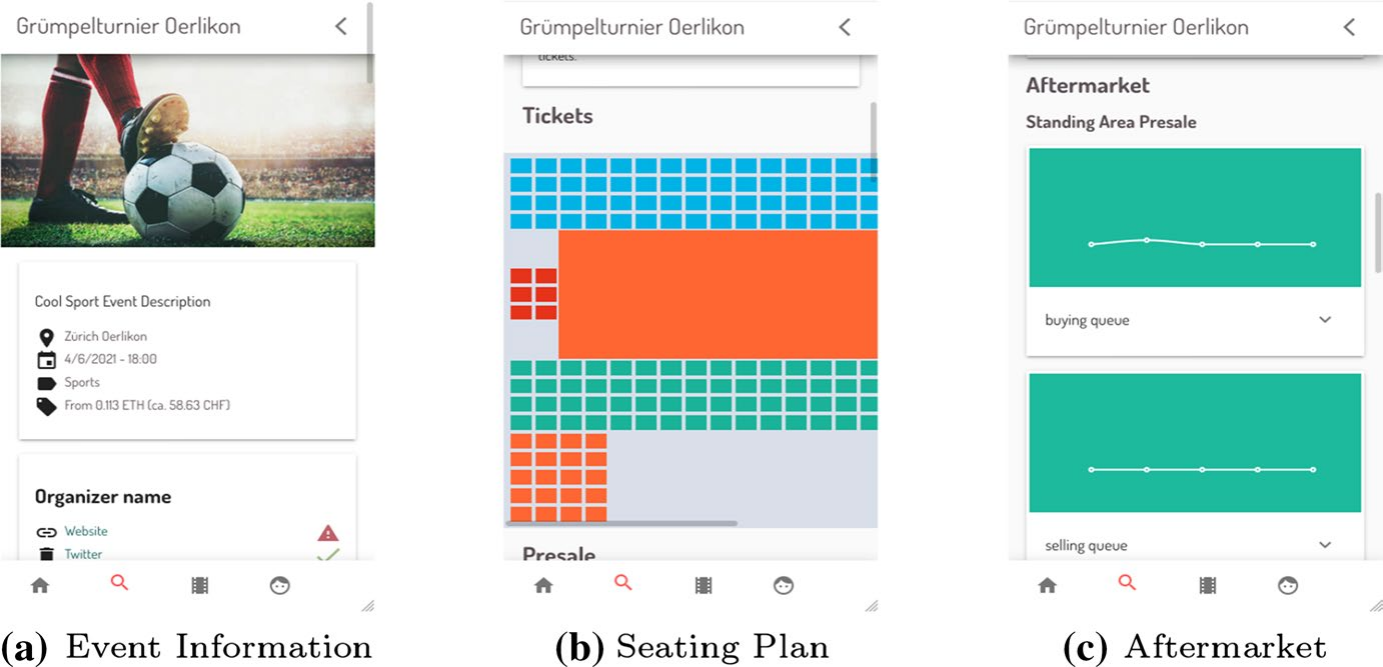
DeTi guest client application views [19]

While DeTi allocates distinct Web applications for hosts, guests, and the access control terminal [19] this paper only covers the guest application. The guest-client Web application is the GUI provided for potential event guests interested in buying or selling tickets. Whereas, the host-client Web application is a tool for event organizers, which allows them to create events on the platform as well as to manage their events including the tickets. Both Apps are built with Vue.js [20], a client-side JavaScript framework [21]. Using the guest client application, guests are able to browse available events and their ticket categories. They are able to connect their Ethereum wallet to the application to enable in-application ticket purchases and message signing. When a user selects an event, she is taken to the event detail page presented in Fig. 17. First presented view (Fig. 17a) is all of the information on the event, as well as an ID-approver chosen by the event host, if any. Furthermore, a user can view a graphical representation of all ticket categories (Fig. 17b). The user can buy any of the available tickets by tapping on the desired category/seat. If the event is still in its *presale phase*, the user can also join the presale from within this view. Lastly, the aftermarket state is shown for each ticket category. For the *buying queues*, the user can directly enqueue or buy an aftermarket ticket if there are offerings in the selling queue.

In order to interact with the Ethereum BC, the Web3.js library is used, which provides the wrapper functionality for data fetching from the BC and invoking remote SC calls. As the storage capabilities of SCs are highly limited, therefore, event-related information (metadata) is stored externally. To keep the architecture as decentralized as possible, the metadata is stored on IPFS. IPFS is accessed from the guest-client application through a JavaScript API.

To reduce the amount of remote calls to the BC and IPFS during application usage, the data is maintained within Vuex, a state management library provided for Vue.js. However, this storage is still in-memory and thus cleared with each application restart. Relying on a solely run-time storage would require a full-fetch of all data on the BC with each application start, which introduces long loading times. To circumvent this, clients use a second, persistent storage, i.e., the indexed Database (IDB); IDB is a schema-less data store supported in most modern browsers which allows storing JSON-formatted data on the client device. The only caveat being that this storage is cleared when the user chooses to remove all browser data.

The guest-client includes a middle layer with wrappers for IDB that handle storage operations and the required logic to only fetch data from the BC when there is something new to display. When the guest-client application is first started up, (called “*Cold Start*"), the SC addresses of all available events are requested from the *Event Factory* SC. From these, the application can retrieve the IPFS hash for the events’ metadata, as well as for all ticket categories of the event. Using the provided hash, the metadata is then requested from IPFS. The guest-client application then builds a JavaScript Data-Object from the received data and stores it in the application state, as well as persists it in the IDB. The stored object contains information such as title, SC address, location, time of the event as well as information on the ticket categories such as price, supply, description, and seat-mapping. In the same way, the guest-client application requests the Ethereum address of the user’s wallet and creates a Data-Object for the user. This also initializes an empty ticket inventory. Each storage entity also contains a record *lastFetchedBlock*, which represents the block number at which it has been updated last, which is set to the current block right before storing them in IDB.

On next application starts, (called “*Warm Starts*"), the IDB is queried for all event contract addresses received from the *Event Factory* SC. If there is an entry for an address present, the application knows that it has already fetched the metadata from the BC. Thus only new events will require a full fetch. However, as aforementioned, the metadata also includes information on the supply of tickets and the owned tickets of the user, which is subject of change. This means that the application needs a way of having the most up-to-date state of the SC at all time. To ensure this, DeTi loads the metadata for all events from their SC and IPFS hash also on warm starts. However, as further discussed in the related evaluations (*cf. *Sect. 5.4.4), this is not feasible due to high loading times even with a low number of events. This problem is solved using Solidity events; SCs can emit events when one of their methods is invoked, and these events contain an identifier (e.g., *ticketBought*), buyer’s Ethereum address, event contract address, and the ticket category. In most cases the client storage can be updated with only the information gathered from these events. These events are indexed with the block number of the BC at the time they were emitted. Hence, on a warm start, the application requests all Solidity events from the *lastFetchedBlock*. For each type of Solidity event, it assigns the correct handler and updates the data models according to the information in the received event.

## Economic Incentives Management in DeTi

**Fig. 18:**
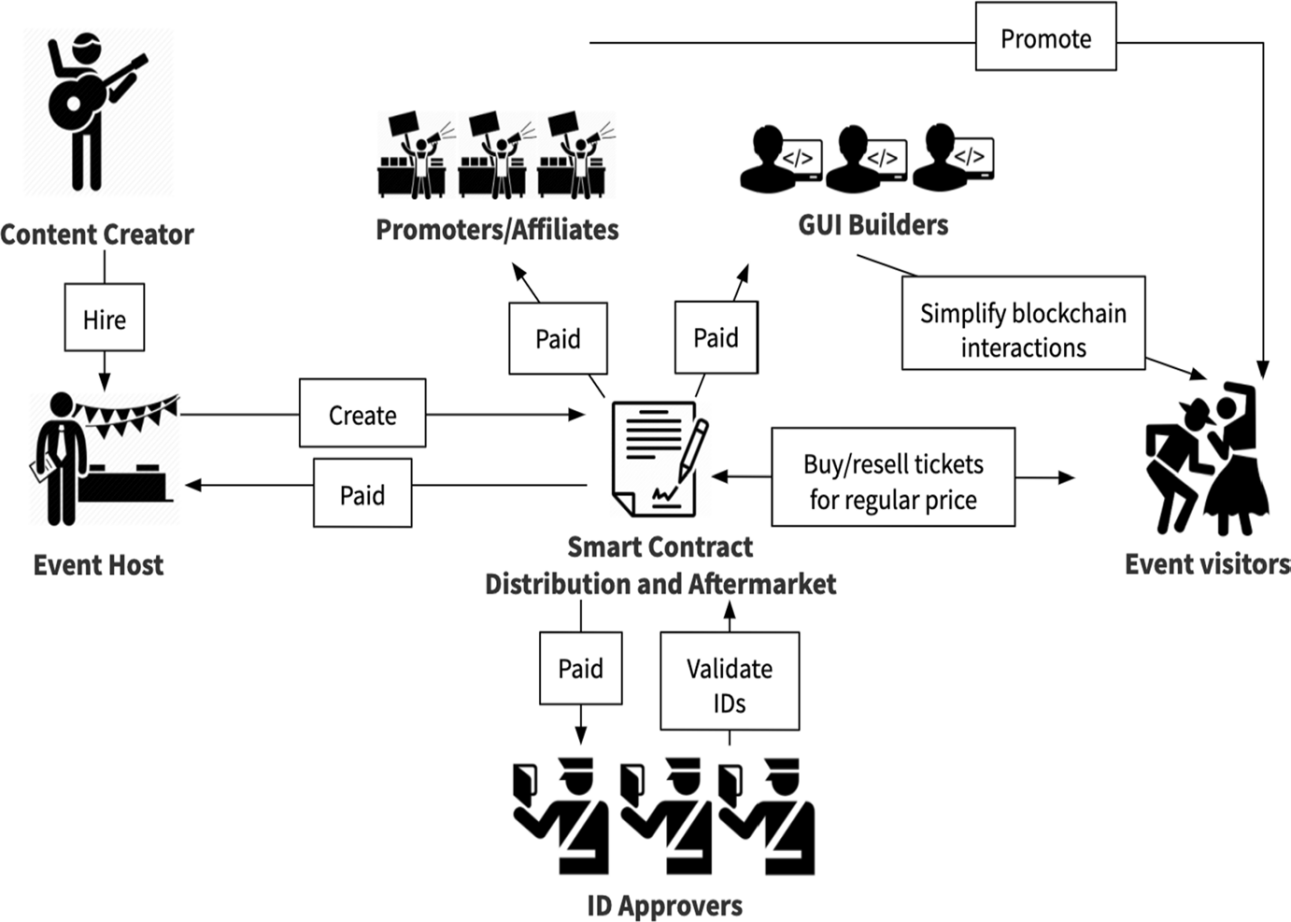
Smart contract-based management of funds flow in DeTi [19]

Stakeholders play crucial roles in the ticketing processes in DeTi. As shown in Fig. 18, DeTi entities are compensated by a shared and transparent economical design that covers all the partners fairly without a regulating entity. This is achieved via automated P2P payments handled by SCs.

A host in DeTi either relies on trustworthy ID approvers or does his own KYC procedure. To incentivize ID approvers to become trustworthy and to compensate them for their service, a fraction of each ticket price is directly paid to the selected ID-approver. Since this is an open design, ID approvers compete with others to become popular among the event hosts.

A GUI builder is also compensated for his service if the ticket is bought through his application. This creates fair competition among different GUI builders. Affiliates are also included in the buying process and are being compensated with direct payment for each ticket that is bought due to their service. DeTi SCs allow passing affiliate addresses and GUI builders as arguments for methods such as buying a fungible ticket. It is important to note that an affiliate has to trust a GUI builder that the affiliate’s address is included in the SC call. However, with the proposed design, it is also in the GUI builder’s interest that other entities redirect guests to their platform. Furthermore, there is no lock-in effect with any of the GUI builders, and they can be replaced if they do not behave trustworthy.

## Evaluation

Evaluation on DeTi is performed on several dimensions including conducting interviews with stakeholders and costumer surveys, calculating SC and TX costs, ID approver cost estimation, and Guest-client approach in data loading/storing [19]. The proof of concept implementation of DeTi has shown that its BC-based design eliminates many of the deficits existing in today’s ticketing industry. A comparative overview of DeTi and studied related work is presented by Table 1.

### Interview and Customer Survey

A feasibility study on DeTi’s approach is performed via interviews by ticketing industry hosts and guests. The interview and proof of concept demo conducted with a system engineer of the “Gurten Festival” [22] —Gurtenfestival is an annual musical festival in Bern, Switzerland [23]—.

Dissatisfaction of Gurten Festival hosts with the current situation of the ticketing industry in Switzerland has driven them to build their own ticketing system to remove middlemen from the process. These middlemen take large cuts and lack integration possibility into their ticketing services. One key issue faced by the Gurten Festival is their presale tickets are usually sold out within a few minute and most of the tickets that appear on the aftermarket are sold for more than the original price. Additionally, their view of personalized tickets, which has changed positively due to the Covid-19 pandemic. Hence, they expect less resistance from the public when it comes to registering the identity or phone number of event guests. Thus, it is foreseen that personalized tickets may be more accepted in the future. supported with the proposed solution.

Further, a customer survey was conducted on potential event guests who attend at least 3 events per year. Results indicate that 41.7% of the participants have already experienced an issue, where they could not buy a ticket due to a slow Internet connection or black market. They also stated that when they searched for tickets on black markets, they only found listings for a higher price than initially offered on the primary market. All the participants would support personalized tickets, as long as the entity that checks the identity was trustworthy. The entities perceived most trustful were government-backed entities, e.g., by a municipality [19].

The proposed idea of a presale was the most controversial concept in the conducted survey. Only 1/3 of the participants preferred a fair presale. They argued, that this was mostly due to the uncertainty a presale brings, and they would have to wait until they know whether they get a ticket or not. Most of them liked it more to just be ready at any given time, since they expected to always get a ticket. The proposed concept of a fair distribution of profit with the GUI-providers, affiliates, and the ID-approvers was received very positively. The proposed model was perceived as being fair. Finally, the biggest problem identified was the core technology used. A lot of participants had no or very limited experience of using cryptocurrencies. Contrarily, people tend to overcome any obstacles to obtain a ticket, depending on the performer artist [19].

### Cost Calculation of SCs and BC Transactions

To minimize the costs, the SCs developed are designed in DeTi such that the number of on-chain TXs can be kept as low as possible. Moreover, an event host can specify which currency (e.g., stable coin) is used for buying tickets. Thus, the host is not exposed to the price fluctuations of the volatility of ETH. As of September 25, 2021, the Ethereum gas (the fee paid by Ethereum users for mining and verifying their TXs) price is 58 Gwei for average TX speed and 1 ETH is worth 2,966 USD [15]. For all the following analysis, these values are used (Table 2).

**Table 2:** Gas cost analysis of smart contracts [19]

Contract	Contract size [kb]	Gas used	Price ETH	Price USD
Library	0.39	139370	0.008	23.5
Identity	1.17	311961	0.018	52.6
Event factory	23.93	5367566	0.311	905.3
Event	21.61	4756013	0.275	802.16

In DeTi a “factory" pattern is applied, i.e., the factory is responsible to create the SC for new events and to keep track of already created events. The *EventFactory* contract is lightweight, but it imports the event contract which holds most of the functionality, and it must only be deployed once. Event contracts are built in a way that the entire event specific functionality is maintained in the *Event* contract. Since deploying a new instance of an SC on Ethereum is expensive DeTi combines all the functionalities for managing fungible as well as non-fungible ticket types in one contract resulting in lower gas costs for an event host. These SCs are designed such that the number of on-chain TXs can be kept as low as possible. Since signing a TX always results in a media break between an application and the wallet, these SCs favor less but expensive TXs over many cheap TXs. As an example, an event host can create multiple ticket types in one TX. Table 3 shows how the gas prices behaves if multiple ticket types are created in one TXs and Table 4 shows how the gas prices evolve if multiple tickets are bought within the same TX. In this scenario, one affiliate is included in the TX to make the price estimations more realistic.

**Table 3:** Cost analysis for creating ticket types within one TX [19]

#Fungible types	#NF types	Gas used	Price ETH	Price USD	Price USD per ticket type
1	0	115656	0.0067	19.5	19.5
2	0	184088	0.01	31.04	15.5
10	0	731625	0.042	123.4	12.34
0	1	115690	0.0039	11.56	11.56
1	1	203322	0.011	34.2	17.2
10	10	819345	0.045	138.2	13.8

**Table 4:** Cost analysis for buying tickets within one TX [19]

#Fungible tickets	#NF tickets	Gas used	Price ETH	Price USD	Price USD per ticket
1	0	117328	0.0068	19.78	19.78
2	0	117328	0.0068	19.78	9.89
10	0	117328	0.0068	19.78	1.97
0	1	145383	0.0084	24.52	24.52
0	2	179586	0.01	30.28	15.14
0	10	453240	0.026	76.44	7.64

Calculated *gas* cost of deploying DeTi’s SCs is as follows. (a) Library SC: 139370 *gas* = 0.008 ETH = 23.5 $, (b) Identity SC: 311961 *gas* = 0.018 ETH = 52.6 $, (c) Event Factory SC: 5367566 *gas* = 0.311 ETH = 905$, (d) Event SC: 4756013 *gas* = 0.275 ETH = 802.16 $. The first three SC costs are paid only once by DeTi owner and the Event SC costs are paid by event host.

To create a fungible ticket type via one TX, 115,656 *gas* is used, equal to 0.0067 ETH or 19.5 $. Whereas, creating a non-fungible ticket via one TX costs almost 11 $. The cost of creating multiple tickets via one TX, e.g., 10 fungible and 10 non-fungible tickets at once, equals 13.8 $. Table 4 represents the costs for buying fungible and non-fungible tickets.

Table 5 shows the same scenario as Table 4 but assuming that ERC20 tokens are being used as the payment method. The TX with ERC20 token is slightly more expensive than using ETH as a payment. However, ERC20 tokens require an additional approval TX in advance which consumes about 44,012 gas (0.002 ETH / 7.42 USD).

**Table 5:** Gas cost analysis for buying tickets with ERC20 token [19]

#Fungible tickets	#NF tickets	Gas used	Price ETH	Price USD	Price USD per ticket type
1	0	163921	0.009	27.64	27.64
0	1	167547	0.009	27.64	27.64
0	2	201750	0.011	34.02	17.01
0	10	475404	0.027	80.18	8.01

As shown in the gas price analysis, a new event is expensive to deploy, but creating ticket types and minting new tickets cost around 1 USD per ticket and type. However, the main benefit from this design is that the host and the guests must sign TXs as seldom as possible. Multiple non-fungible tickets of different types can be minted within the same TX. This concept is applied to all the TXs in the ticket distribution, presale, as well as the aftermarket. One can create multiple presales with one TX. Also, it is possible to create a buy order for multiple fungible as well as non-fungible types within a single TX. Thus, the user experience is more like a traditional application where a customer only has to sign a TX when the shopping cart is checked out.

The functionality of using other currencies such as stable coins are integrated in the contract by default. Every value transfer on the contract will be made in the selected currency by the event host. TXs become slightly more expensive as shown in Table 5 and require an additional approval TX in the ERC20 contract. However, it enables the host and guests to transact with less volatile currencies.

### Cost Estimation of ID Approver

One of the considered aspects in the design and implementation of the ID approver was the amount paid for an ID approver to verify a potential guest. As the ID approver stores the proofs to the BC, TX costs are paid by the ID approver. The ID approver also has to pay for the third-party services used to verify a guest’s identity. The ID approver is compensated, whenever a guest, that has been verified by him, buys a ticket. If, for example, all the affiliates together were compensated with 10% of the ticket price. Therefore, the amount received by the ID approver decreases when the number of affiliates for this ticket sale increases. For this evaluation, it is assumed that there are only two affiliates besides the ID approver, rewarding him with 3.3% of the ticket price.

Email: Assuming that an ID approver already has an existing email server, the cost for an additional email address is negligible. Therefore, only the gas cost of the BC TX is taken into account. The function to approve a guest consumes a total amount of 28,845 *gas* equal to a cost of 0.00098 ETH, i.e., 0.37 $. This means, that the ID approver is already paid for his expenses, once the guest bought one ticket that cost more than 12 $.

Phone: The cost of sending text messages via a service provider like Twilio is 0.069 $ per SMS sent and 8 $ per phone number per month [24]. With a pessimistic assumption of only 10 verifications happening by an ID approver per month, the total cost equals 0.869 $ per text message. This results in a total cost of $1.239, which means that the ID approver has already compensated for his expenses, once the guest bought one ticket that costs more than 41 $.

Know-Your-Customer (KYC): The cost of AWS recognition service per image reduces with the number of pictures analyzed increases, resulting in an average of 0.0012 $ per image [25]. Overall, this results in a total cost of 0.3712 $, which means, that the ID approver is already compensated, once the guest boys one ticket at a cost of more than 12 $.

### DeTi Efficiency in Fetching Blockchain Data

A crucial aspect of any BC-based application lies within the responsiveness in user interactions. Hence, DeTi’s guest client application provides an interface that is never blocked for user interaction, except when the user chooses to switch to another cryptocurrency wallet. The following section discusses some performance benchmarks, along with their respective impact on the usability of the DeTi application, and overviews the measures taken for improvements in certain benchmarks. All benchmarks have been evaluated on an Intel i5-7600 CPU with a clock speed of 3.5 GHz and 4 individual cores.

#### Baseline—Event Loading

**Fig. 19:**
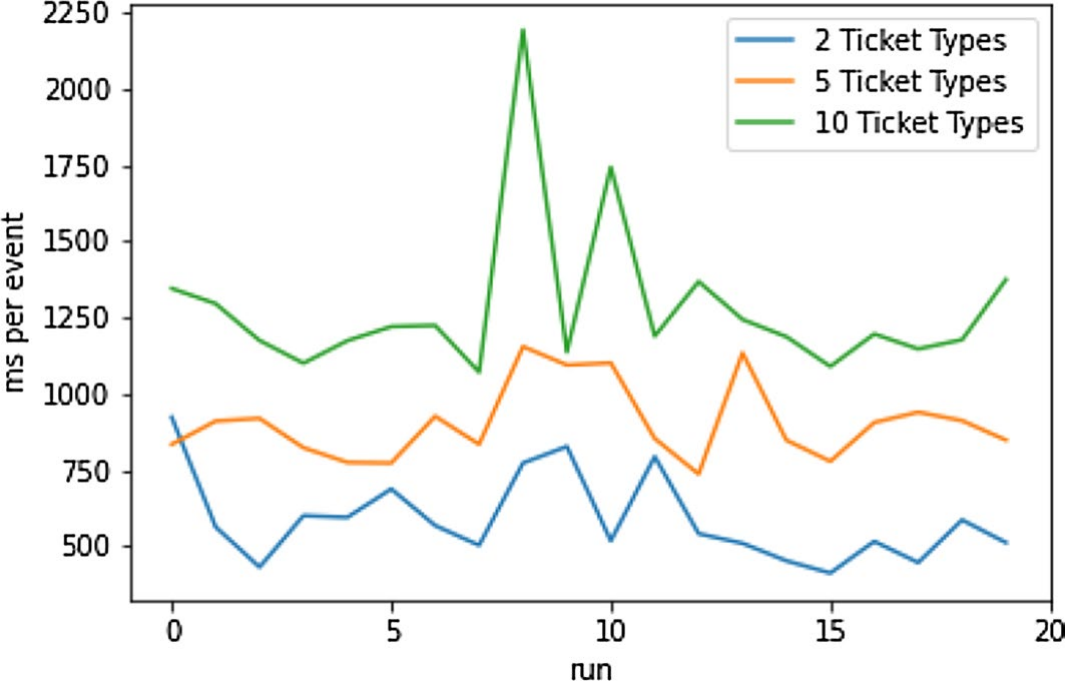
Baseline event loading times [19]

In order to get a solid baseline for event loading times, the application was tasked with fetching event data for three different events, with two, five, and ten ticket types (TT) each. For this experiment, no caching mechanism has been used (*cold start*). The loading times are shown in Fig. 19. A higher number of ticket types leads to consistently longer loading times, when all other variables such as the number of Solidity events are isolated. Table 6 shows mean and average loading times over the course of 20 consecutive runs. The median is consistently lower across this and all following experiments. From Table 6 one can extrapolate a difference of approximately 320 ms in loading times when going from two to five ticket types. The absolute increase in time is slightly lower when going from five to ten ticket types.

**Table 6:** Baseline event loading time statistics [19]

	Mean [ms]	Median [ms]
2 TT	587.03	551.39
5 TT	904.23	878.72
10 TT	1281.94	1191.82

#### Baseline—User Interactions 

**Fig. 20:**
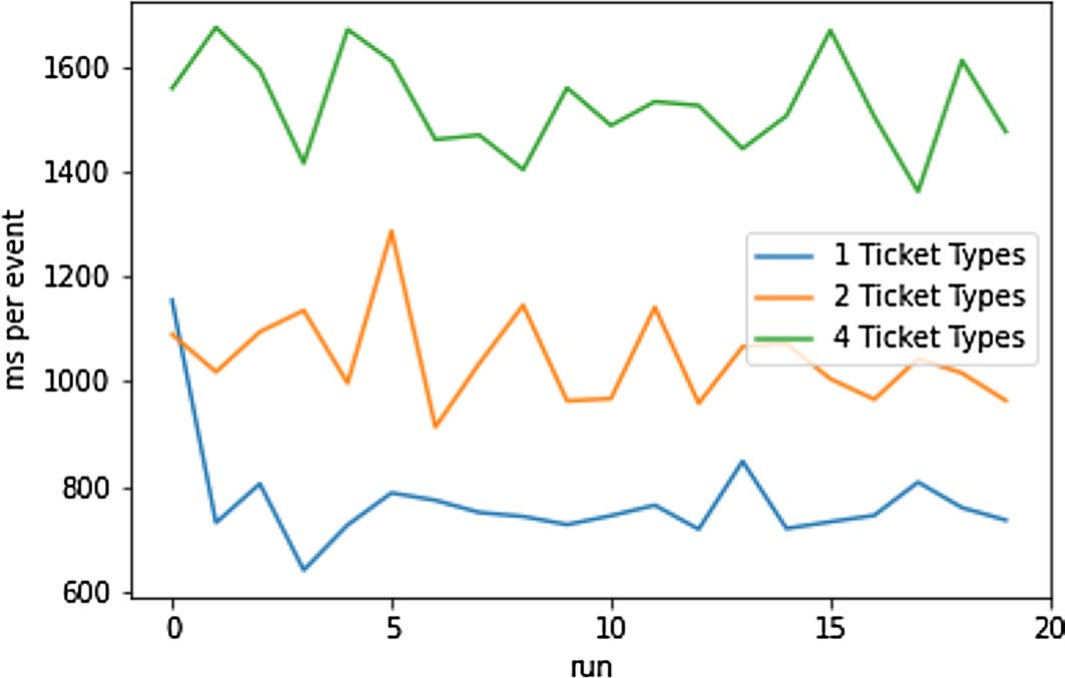
Baseline user interactions [19]

The second baseline was measured in order to capture the effect of user actions. For this, one event with one, two, and four ticket types respectively was deployed; 50 user accounts funded with test Ethers, each of which bought a ticket and made an aftermarket listing. Figure 20 shows the loading times for this scenario. The loading times were higher compared to Fig. 19. This behavior is expected due to the moderate amount of user induced Solidity events. Table 6 presents the mean and the average loading times over in 20 runs (Table 7).

**Table 7:** Baseline user interaction loading times statistics [19]

	Mean [ms]	Median [ms]
1 TT	770.80	744.55
2 TT	1043.78	1026.26
4 TT	1526.70	1515.72

#### Account Switching

**Fig. 21:**
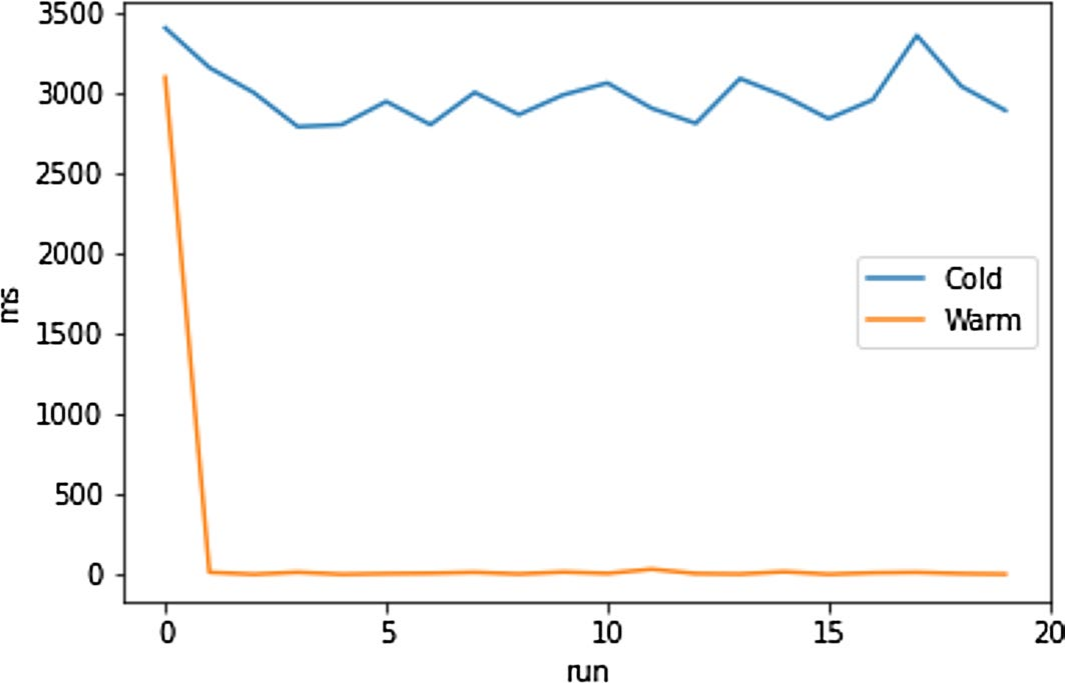
Ethereum user account switching times [19]

One feature specifically enabled in DeTi by using an IDB is the immediate user account switching. Figure 21 shows an experiment on a BC state with 20 events deployed. One user account was configured to buy a ticket for each of these events and to make an aftermarket listing as well. On a cold start it took around three seconds to load a user’s data, which although covered with a loading screen, is a notable loading time. The warm start, i.e., using the locally cached data from the abstraction layer, was able to consistently load all the user’s tickets and display them within around 0.2 seconds. Which clearly indicated the design decisions taken in DeTi are well enhance its performance (Table 8).

**Table 8:** Ethereum user account switching times statistics [19]

	Mean [ms]	Median [ms]
Cold	2978.96	2963.11
Warm	164.99	8.98

#### Pre-loading in Detail

**Fig. 22:**
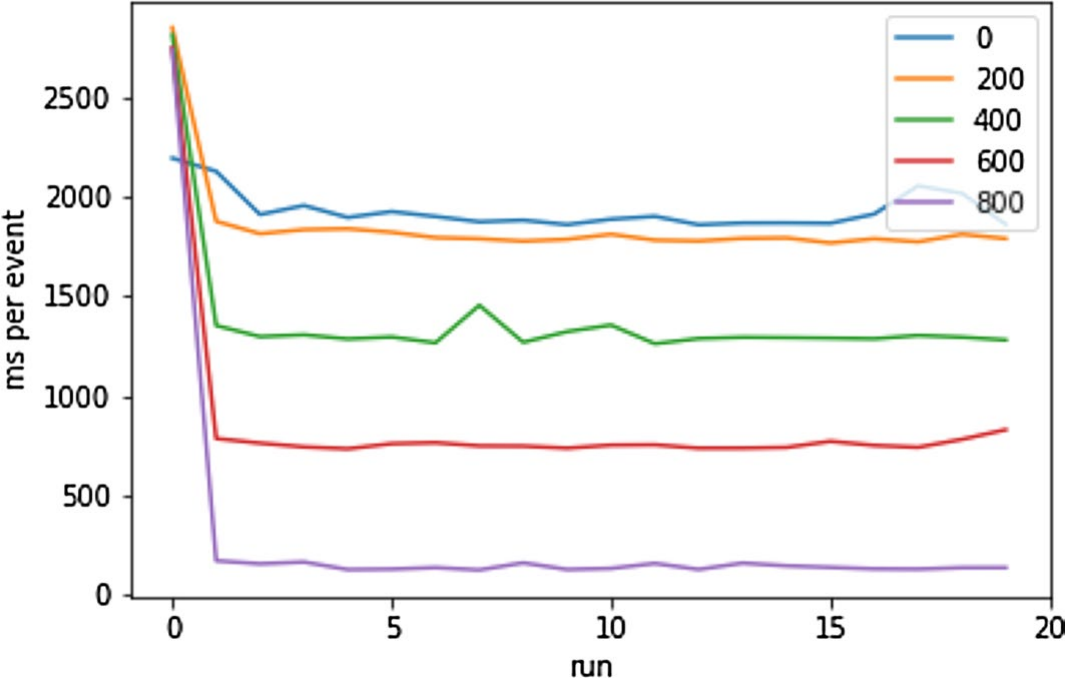
User switching times [19]

In this last evaluation, an event with numerous user interactions over the course of 858 blocks was prepared on the BC. The guest application was tasked with completely fetching the events’ data while having the information from all Solidity events up to increasing block number cached in the abstraction layer. Figure 22 shows the loading times for each level of cached data. The labels show the block number up until which the data was cached (lower means more data to load). Block 858 was the last block with new Solidity events. Table 9 shows the mean and average loading times for each stage. The benefit of local caching and an efficient data abstraction manifests itself in this evaluation. With 50% of the data stored locally, the loading time decreases almost linearly and with over 90%, the application can load an event’s data within a fraction of a second.

**Table 9:** User switching time statistics [19]

Starting block	Mean [ms]	Median [ms]
0	2978.96	1900.95
200	1933.94	1794.26
400	1855.97	1294.84
600	1381.26	750.0
800	268.80	133.41

### Addressing Identified Security Risks by DeTi

Based on the discussions provided above, a summary of the measures taken by DeTi in addressing the identified security concerns in Sect. 2.3 is listed in Table 10. Unfortunately, assessing related work against all the mentioned security risks is not possible due to lack of documentation or accessibility to the details of those systems’ implementations. However, a key finding of the studies in this work revealed similar inefficiencies in related work. That is, except for the Origin protocol, –which is not supporting event ticketing management–, other studied related work are failing in allowing users to buy a ticket with a BC wallet and to link that ticket to the user identity such that the ticket cannot be resold on a black market. Moreover, the studied related work propose deposit-based methods to prevent fraudulent events, which is different from the novel social trust-based solution in DeTi.

**Table 10:** Summary of measures taken in DeTi to address identified security concerns [19]

Security risk	DeTi solution	Related work solutions
Front running	Developing a lottery style for presale	Not prevented
Random number manipulation	Employing block hash	Multiple approaches
Ticket invalidation	Smart Contract-based which requires identification	Not offered since ticket-to-identity binding is not happening on chain
Identity fraud	KYC, ID-Approvers, Social Trust	Offchain and KYC-based
Fraudulent events	Social proofs and trust certificate	Deposit-based
Event spamming	Prevented as Ethereum TX gas fees are paid by users	Not available
Black market	Preventing from reselling tickets with higher prices than original price	Not prevented

## Summary and Discussion

DeTi addresses the requirements identified in Sect. 2 by the integrated and tightly coupled aftermarket logic. DeTi provides a novel function that is not available on ticketing platforms as of today. The proposed presale design guarantees a fair distribution if the demand for an event is higher than its supply. The lottery approach is transparent and publicly available. Furthermore, the presale logic prevents front running attacks and will not lead to sudden surges in gas prices. Furthermore, both the question of validity when buying a ticket from another user, and the inflated prices on tickets with high demand are addressed. The queuing-based architecture of aftermarket enables buyers and sellers to transact with each other. Also, there is no trust needed when exchanging a ticket on the aftermarket. A seller cannot trick buyers by not sending the ticket after receiving the money, since the SCs used guarantee that the ownership is only transferred if the correct amount of money is sent.

The architecture of the DeTi benefits all stakeholders in the system. Event guests profit from fair ticketing pricing, even on the aftermarket. Tickets cannot be sold for higher prices than the original cost of a ticket, since ticket scalpers have no scope of action and are prohibited on the protocol level. Event guests do not need to check whether a ticket for the same event is offered on any other platform, since they all connect to the same data. To prohibit scalpers from misusing the aftermarket DeTi verifies the identity of the guests and only verified accounts can buy tickets from the SC. This crucial part of identifying and approving the identity of a guest and linking the identity to an Ethereum address is done by the ID approver. As the identity SC is used to store the identities, every entity may act as an ID approver. Moreover, social trust certificates used in DeTi to create a layer of trust that is higher than in current online ticketing platforms without the need of a trusted third party. Aggregating ownership proofs across multiple social profiles in the event listing increases the legitimacy of an event. DeTi creates a new market for ID approvers, who are needed in a decentralized system, since there are no restrictions from creating a wallet and interacting with the platform. ID approvers are incentivized to act trustworthy since they are financially compensated if they are chosen by the event hosts.

As part of the future work, it is foreseen to reduce the fees for creating new instances of the same contract by using the proxy pattern e.g., as introduced in EIP-1167 [26]. In addition, an analysis of loading times for the performance evaluation of the client-side caching mechanism when deployed on an Ethereum main net can provide real world verification of the DeTi’s performance, which is deemed worthy to consider as a future work. Finally, moving forward in adapting DeTi to newer generations of BCs such as Eth 2.0, employing a randomness models such as Beacon chain is one potential way to be considered.

## References

[1] Davies, R.: Viagogo condemned over ed sheeran cancer benefit concert tickets. https://www.theguardian.com/money/2017/feb/17/viagogo-condemned-ed-sheeran-cancer-benefit-concert-tickets-teenage-cancer-trust (2017). Accessed 25 Sep 2021

[2] Scheid, E.J., Rodrigues, B., Killer, C., Franco, M., Rafati Niya, S., Stiller, B.: In: Goedicke, M., Neuhold, E., Rannenberg, K. (eds.) Blockchains and Distributed Ledgers Uncovered: Clarifications, Achievements, and Open Issues. IFIP AICT Festschrifts, pp. 1–29. Springer, Cham (2021)

[3] Hosseini Bamakan SM, Motavali A, Babaei Bondarti A (2020). A survey of blockchain consensus algorithms performance evaluation criteria. Expert Systems with Applications.

[4] Dhillon V, Metcalf D, Hooper M (2021). Unpacking Ethereum.

[5] Hafid A, Hafid AS, Samih M (2020). Scaling blockchains: a comprehensive survey. IEEE Access.

[6] Liu, M., Fraser, J.: Origin Protocol. https://www.originprotocol.com/en/whitepaper (2019). Accessed 25 Sep 2021

[7] GET Foundation Team: Guaranteed entrance token smart event ticketing protocol. https://get-protocol.io/files/GET-Whitepaper-GUTS-Tickets-latest.pdf (2017). Accessed 25 Sep 2021

[8] Blockparty: Blockparty: an event ticketing blockchain protocol. https://cms.goblockparty.com/wp-content/uploads/2019/04/Blockparty-Event-Ticketing-Whitepaper-v-4.6.pdf (2018). Accessed 25 Sep 2021

[9] Aventus: a layer-2 blockchain protocol that brings scalability, lower costs, and speed to ethereum transactions. https://www.aventus.io/. Accessed 25 Sep 2021

[10] Mathieu, F., Mathee, R.: Blocktix: decentralized event hosting and ticket distribution network. https://whitepaper.io/document/332/blocktix-whitepaper (2017). Accessed 25 Sep 2021

[11] Regner, F., Urbach, N., Schweizer, A.: NFTs in practice–non-fungible tokens as core component of a blockchain-based event ticketing application. In: Fortieth International Conference on Information Systems, Munich (2019)

[12] Li, X., Niu, J., Gao, J., Han, Y.: Secure electronic ticketing system based on consortium blockchain. In: KSII Transactions on Internet and Information Systems (TIIS), vol. 13, pp. 5219–5243. Korean Society for Internet Information (2019)

[13] Lin, K.-P., Chang, Y.-W., Wei, Z.-H., Shen, C.-Y., Chang, M.-Y.: A smart contract-based mobile ticketing system with multi-signature and blockchain. In: 8th Global Conference on Consumer Electronics (GCCE 2019), Osaka, Japan, pp. 231–232 (2019). IEEE

[14] How IPFS Works. https://docs.ipfs.io/concepts/how-ipfs-works (2020). Accessed 25 Sep 2021

[15] Ethereum average gas price chart. https://etherscan.io/chart/gasprice. Accessed 25 Sep 2021

[16] Plasma. https://docs.ethhub.io/ethereum-roadmap/layer-2-scaling/plasma/. Accessed 25 Sep 2021

[17] Engage customers on any channel, any time. https://www.twilio.com/. Accessed 25 Sep 2021

[18] Service, A.W.: Amazon rekognition. https://aws.amazon.com/de/rekognition/. Accessed 25 Sep 2021

[19] Bachmann, S., Bucher, M., Brasser, C., Spielmann, N.: Design and prototypical implementation of blockchain ticketing, Zürich, Switzerland (2021). Communication Systems Group, Department of Informatics. https://owncloud.csg.uzh.ch/index.php/s/RBFy4sJo39ZxrSa

[20] Vue.js: The progressive JavaScript framework. https://vuejs.org/. Accessed 25 Sep 2021

[21] Simon, B., Brasser, C., Bucher, M., Spielmann, N.: Blockchain ticketing. https://github.com/bc-ticketing. Accessed 25 Sep 2021

[22] GRTN22. https://gurtenfestival.ch/en/. Accessed 25 Sep 2021

[23] Gurten festival. https://en.wikipedia.org/wiki/Gurtenfestival. Accessed 25 Sep 2021

[24] Twilio: SMS pricing. https://www.twilio.com/sms/pricing/ch. Accessed 25 Sep 2021

[25] Amazon rekognition pricing. https://aws.amazon.com/de/rekognition/pricing/. Accessed 25 Sep 2021

[26] Murray, P., Welch, N., Messerman, J.: EIP-1167: minimal proxy contract. https://eips.ethereum.org/EIPS/eip-1167. Accessed 25 Sep 2021

